# Organoids in respiratory virus research: advances and perspectives

**DOI:** 10.1186/s43556-025-00343-x

**Published:** 2025-11-06

**Authors:** Xingling Li, Haiqing Xiao, Ming Zhou, Chuanlai Yang, Xinyi Yang, Tong Cheng, Lunzhi Yuan, Ningshao Xia

**Affiliations:** State Key Laboratory of Vaccines for Infectious Diseases, National Institute of Diagnostics and Vaccine Development in Infectious Diseases, Xiang An Biomedicine Laboratory, School of Life Sciences & School of Public Health, Xiamen University, Xiamen, Fujian China

**Keywords:** Human organoids, Respiratory viruses, Immunopathology, Drug discovery

## Abstract

The pandemics of respiratory viruses pose a worldwide public health problem and bio-safety threat. Therefore, the development of high-throughput and accurate infection models is crucial for elucidating viral pathogenesis and accelerating countermeasures to address the evolving respiratory viruses and the unexpected outbreaks of emerging variants. Compared to traditional 2D cultures, organoids exhibit pronounced intercellular interactions, extracellular matrix signaling, and tissue-specific multicellular cooperation, thereby more accurately recapitulating the in vivo microphysiological environment. However, research involving animal models typically requires prolonged experimental timelines, making it challenging to perform high-throughput screening or rapidly develop therapeutic strategies within the valuable timeframe. Since the outbreak of SARS-CoV-2, organoids have significantly advanced basic virology research and demonstrated potential in replicating the pathological and immunological characteristics in human patients. This review provides a comprehensive summary of the theoretical foundations, methodological framework, and complete procedures for identification and validation in organoid construction, along with their applications in the investigation of various respiratory viruses, such as coronaviruses, the influenza virus, respiratory syncytial virus, and others. Overall, the development of organoids, in conjunction with the integration of interdisciplinary technologies, has significantly advanced our fundamental understanding of the immunopathology process of respiratory viral infections, improved research efficiency, and provided precise tools for translational medical research.

## Introduction

The emergence of the severe acute respiratory syndrome coronavirus 2 (SARS-CoV-2) pandemic has resulted in billions of coronavirus disease (COVID-19) cases and more than six million deaths worldwide, continuously threatening public health security (https://coronavirus.jhu.edu/map.html). Meanwhile, the ongoing epidemics of other important respiratory viruses, such as influenza and respiratory syncytial virus (RSV), continue to cause high severity and mortality, particularly in vulnerable populations such as infants, pregnant women, the elderly, leukemia patients, and organ transplant recipients [1]. Unfortunately, both SARS-CoV-2 and influenza viruses mutate over time, giving rise to variants with increased infectivity and immune evasion capabilities, often leading to breakthrough infections even in fully vaccinated populations [2–4]. Moreover, current clinical treatments and capacity are inadequate to address severe pneumonia, acute respiratory distress syndrome (ARDS), multiple organ failure and mortality caused by respiratory viral infections [5–8], which necessitates a critical need for high-throughput, accurate and accessible models that closely mimic human tissue characteristics for the investigation of pathology mechanisms and the development of novel countermeasures.

Traditional animal models have played a critical role in basic and translational research on respiratory viruses, particularly in the preclinical evaluation of vaccines and drugs [9, 10]. Nevertheless, the large-scale implementation of animal models in addressing public health crises caused by highly pathogenic pathogens is significantly constrained by methodological limitations, ethical concerns, and translational uncertainties. In particular, research on respiratory viruses leading to widespread transmission and severe sequelae encounters substantial challenges due to the stringent requirements of Biosafety Level 3 laboratory environments. Although 2D monocellular culture systems exhibit cost-effectiveness, experimental scalability, and operational expediency in modelling highly pathogenic respiratory viral infections [11], their scientific utility remains constrained by inherent biological limitations. These systems exhibit monotypic cellular homogeneity, deficient extracellular matrix(ECM) integrity, and abnormal cytoarchitectural polarity. Fundamentally, they lack physiologically accurate 3D histoarchitecture, multicellular signalling networks, and in situ immunometabolic microenvironments, thereby contributing to systematic epigenetic perturbations, organotypic functional deviations, and compromised therapeutic response predictability [12]. Therefore, developing in vitro platforms that accurately represent the virological, pathological, and immunological characteristics of respiratory viruses is essential for improving the simulation of research models and minimizing dependence on animals.

The development of in vitro 3D organoids has recently achieved significant advancements in respiratory virus research. Organoids, generated through the self-organisation of stem cells, recapitulate original biological features such as intercellular interactions, ECM signalling, and tissue mechanical properties, thereby closely mimicking the in vivo cellular microenvironment [13]. When utilised for the construction of infection models, organoids exhibit high levels of similarity and maintain individual genetic information, thereby revealing human-specific host–pathogen interactions. This approach bridges the gap between in vitro models and in vivo research, streamlines the research process associated with animal models, and enhances accessibility for future investigations into respiratory viral infections.

This review aims to summarize recent advances and applications of organoids in fundamental and translational studies of important respiratory viruses, including influenza, RSV, and SARS-CoV-2. We outline the advantages and limitations of current organoid models and discuss new directions for improving the efficiency of organoid generation and functional optimization. Furthermore, we provide unique insights and perspectives for expanding the application scenarios of organoid models for the discovery and evaluation of next-generation vaccines and drugs against respiratory virus infections.

## Generation of organoids

Organoids are self-organising, expanding 3D cultures derived from stem cells [14]. These in vitro systems recapitulate native tissue architecture through self-organisation, exhibiting spatial patterning of functionally specialised cell lineages and thereby preserving the physiological features and biological functions of primitive tissues or organs [15, 16]. Consequently, organoid technology is driving transformative advancements in virology research, revolutionizing disease pathogenesis modelling, accelerating antiviral therapeutic discovery, and enabling patient-tailored treatment strategies [17]. However, the application requires acknowledgement that this technology stems from interdisciplinary integration. Achieving its potential necessitates dynamic adaptation during construction, coupled with rigorous morphological and functional validation, to fulfil the requirements of virological studies and therapeutic strategy development.

### Origin and development of organoids

The origins of organoid technology can be traced back to 1910, when H.V. Wilson demonstrated that mechanically detached sponge cells could autonomously organise into new, fully functional sponge organisms [18]. The groundbreaking study revealed a significant insight that adult organisms could give rise to new organisms independently, without external assistance or beginning at a specific anatomical stage. However, the investigation encountered challenges related to the viability of the tissue post-cell fusion, leading to a temporary cessation of further research. From 1950 onwards, numerous research groups attempted to replicate Wilson's methodology to investigate whether higher animals could also undergo self-organisation. By shearing tissues and allowing them to regroup and self-organise, similar outcomes were achieved [19, 20]. These experiments demonstrated that isolated tissues could develop into organoids with distinct shapes and functions through self-organisation, distinguishing organoids from other 2D or 3D culture techniques. The early phases of 3D cell culture, which commenced in 1965, defined organoids as aberrant cell growth or multicellular structures. In 1975, James Rheinwald and Howard Green proposed a technique for reconstructing 3D tissue structures from cultured human stem cells [21]. In 1977, Emerman and Pitelka cultured mammary epithelial cells for one month using floating collagen gels that expressed lactic proteins, marking a significant milestone in 3D culture methods [22].

Stem cell research was initiated in the late twentieth century with the pioneering in vitro culture of embryonic stem cells (ESCs) from mouse embryos in 1981. Mesenchymal stem cells were subsequently discovered by A.J. Friedenstein in 1987, followed by the isolation of human ESCs for the first time by American biologist James Thomson in 1998 [23]. In 2007, Professor Thomson achieved a significant milestone by successfully generating induced pluripotent stem cells [24]. Another significant advance was made in 2009 when Hans Clevers' laboratory successfully utilised a single murine LGR5 + intestinal stem cell to self-organise in vitro into an intestinal organoid with a crypt-villus structure [16]. Since then, researchers have broadened their horizons, successfully cultivating the brain, liver, kidney, pancreas, and other organoids from human pluripotent stem cells(PSCs). Overall, the rapid progress in stem cell research has revitalised organoid research endeavours.

Building upon these foundational technologies, modular induction frameworks were established, which integrate biochemical cues, biomechanical forces, and computational modelling to guide stem cell self-organisation into anatomically patterned organoids.

### Induction culture and establishment of organoids

**Fig. 1:**
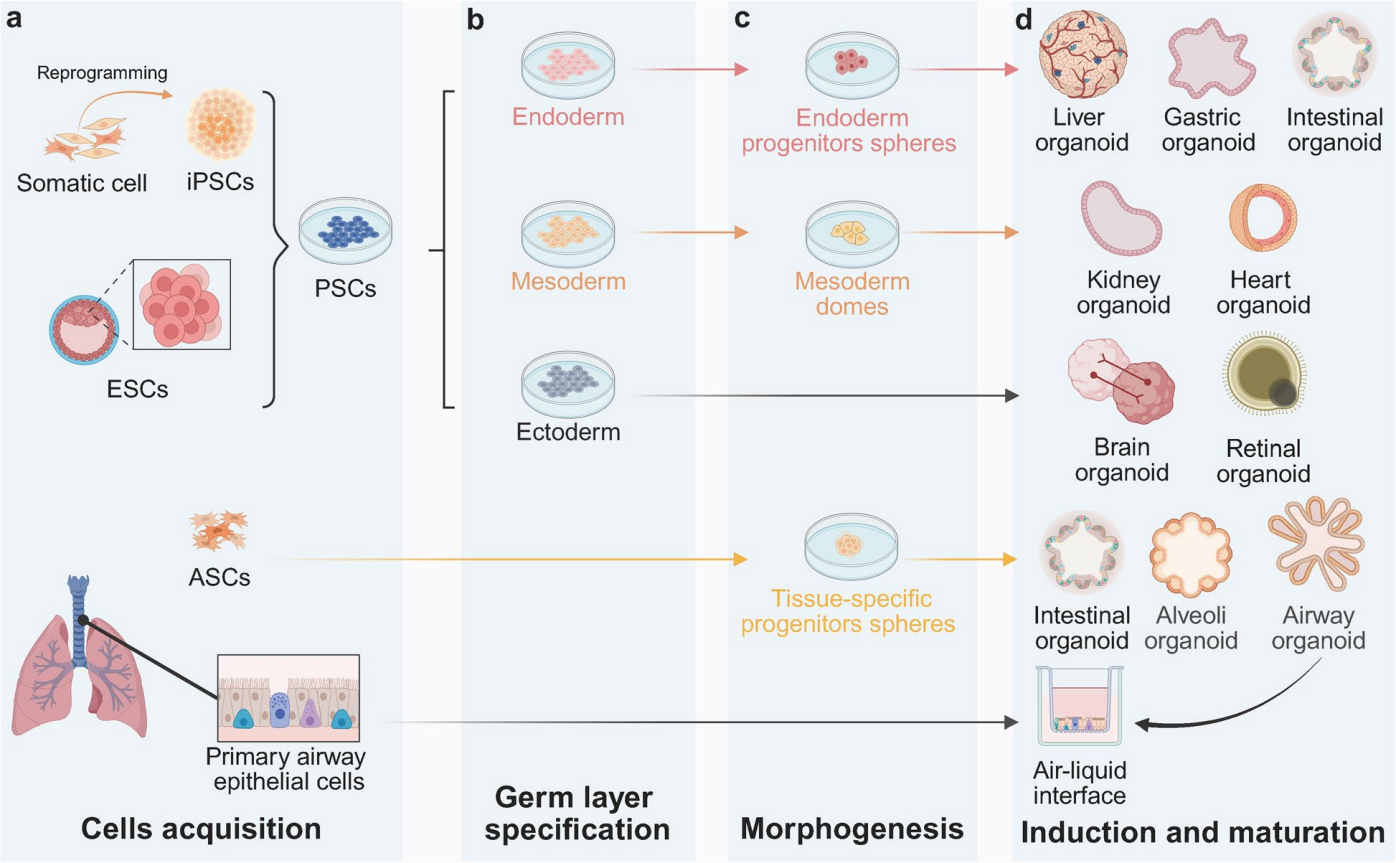
Cell source of organoids (**a**) ASCs are typically obtained from tissue biopsies; iPSCs are generated through the reprogramming of somatic cells such as fibroblasts; ESCs are isolated from the inner cell mass of the blastocyst-stage embryo; and mature organoids or primary airway epithelial cells obtained from tissue biopsies can be used as cell sources for rapid ALI culture. **b**, **c** Both iPSCs and ESCs belong to PSCs that can initially be directed toward specific germ layers and subsequently further differentiated into organ-specific progenitor cells, whereas ASCs can be directly differentiated into progenitor cells. **d** Progenitor cells cultured in a 3D environment and induced to self-organise ultimately forming organoids. (Created in https://BioRender.com)

**Fig. 2:**
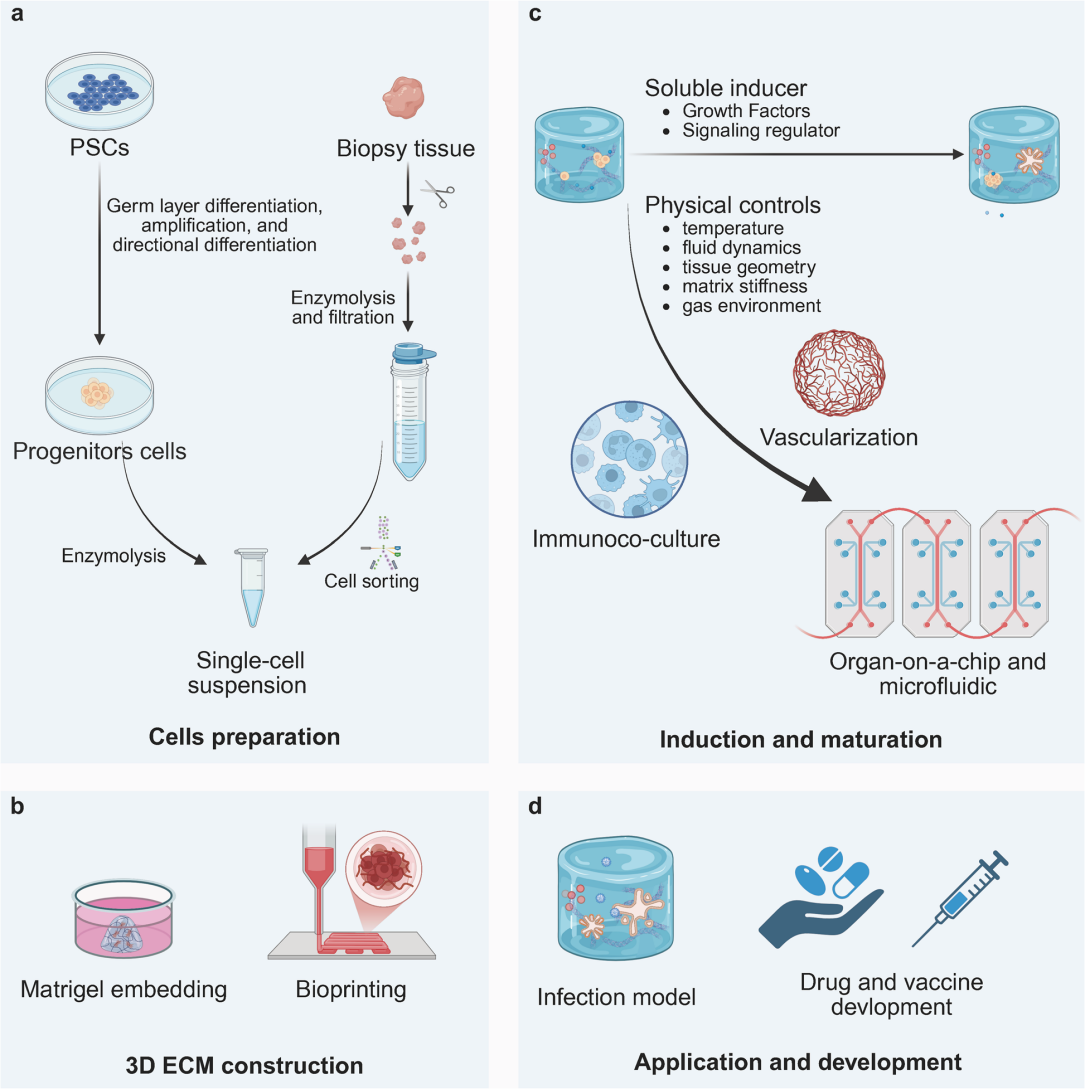
The process of organoid construction (**a**) Following mechanical separation and enzymatic digestion, the biopsy tissue was subsequently processed to generate a single-cell suspension, while the PSCs underwent germ layer differentiation, pre-expansion, and directional differentiation into progenitor cells. **b** The single-cell suspension is seeded into a specific ECM by matrixgel or bioprinting. **c** Following appropriate induction and differentiation, organoids progressively attain maturation. Through further optimization—such as physical controls, immune regulation, and vascular perfusion—combined with microfluidic technology, highly mature organoids can be cultivated on a chip. **d** Mature organoids can be utilized to establish infection models as well as to develop drugs and vaccines. (Created in https://BioRender.com)

The construction of organoids typically involves bioprinting or matrigel embedding to inoculate stem cells within specific scaffolds [25–27], subsequently induced to self-organise into 3D structures, with particular cytokines or dynamic physiochemical stimulation [28, 29] (Figs. 1 and 2). The process conventionally comprises several critical stages: sample acquisition and pretreatment, tissue digestion and preparation of single-cell suspensions, stem cell sorting, inoculation and initial culture, expansion and passaging, identification, and application analysis.

#### Cell source for the generation of organoids

In general, the cell sources utilised for organoid-based virus research include PSCs, adult stem cells (ASCs), or tissue-derived primary cells [30, 31]. PSCs encompass both ESCs and induced pluripotent stem Cells (iPSCs) [30] (Fig. 1 a). PSCs can initially be directed toward specific germ layers and subsequently further differentiated into organ-specific progenitor cells and eventually organoids (Fig. 1 b, c, and d). ESCs exhibit unlimited proliferation potential and capacity for standardised cultivation. Within the respiratory system, for instance, ESCs can differentiate into various types of respiratory epithelial cells, including ciliated cells, goblet cells, and basal cells, thereby enabling the construction of airway or alveolar organoids [32]. Despite the utilisation of well-established ESCs cell lines in these studies, ethical concerns remain. Moreover, the complexity of the differentiation process and variability among different cell lines limit their practical application [33]. iPSCs are typically generated by reprogramming patient-derived somatic cells, such as skin fibroblasts, thereby alleviating the ethical concerns [34]. The preservation of the donor's genetic background facilitates the development of personalised organoids, which potentially enhance the investigation of genetic influences on viral susceptibility [35]. Therefore, challenges persist in optimising differentiation efficiency and managing the substantial costs involved. Yamamoto et al. successfully generated and achieved long-term expansion of alveolar organoids using SFTPC + alveolar stem cells derived from iPSCs [36]. These cells demonstrate robust self-renewal capabilities, while their morphology, transcriptome, differentiation process, and cellular heterogeneity closely resemble those of in vivo developing alveolar type 2 cells (AT2). In summary, iPSCs can not only be utilised for personalised organoids to elucidate individual variations in infection susceptibility but also facilitate high-throughput drug screening and development via standardised organoid-based viral infection platforms.

ASCs constitute a class of undifferentiated cells found in mature tissues or organs, which possess self-renewal capabilities and multidirectional differentiation potential, playing a crucial role in tissue maintenance, repair, and regeneration [37], and can be directly differentiated into progenitor cells (Fig. 1 c). For instance, respiratory or alveolar epithelial stem cells derived from nasopharyngeal, bronchial, or alveolar biopsy tissues can be utilised to construct airway and alveolar organoids [38, 39]. These organoids preserve the natural tissue architecture and cellular diversity, including ciliated cells, goblet cells, and club cells, while maintaining the genetic background of donors and the epigenetic characteristics of the original tissues or organs [40] (Fig. 1 d). This approach enhances differentiation efficiency and reduces culture costs [41, 42], making it particularly suitable for personalised infectious research.

Primary airway epithelial cells cultured at the air–liquid interface (ALI) have been successfully employed to establish stratified epithelial structures that closely recapitulate physiological conditions (Fig. 1 a and d). This model is particularly suitable for investigating viral transmission mechanisms and can rapidly simulate the SARS-CoV-2 invasion process [43, 44] and evaluate mucosal immune responses induced by nasal spray vaccines [45]. However, due to the gradual loss of differentiation capacity in primary cells, the long-term culture of this system presents significant challenges. Consequently, it is essential to periodically replenish the cell source and, if necessary, enhance the culture system to ensure prolonged stability. Fortunately, the methodology for constructing ALI systems utilising well-established organoids has become increasingly standardised [46–48]. Meanwhile, this system could maintain the genetic integrity of the original tissue while effectively addressing the limitations inherent to primary cell cultures [49, 50].

#### Cell preparation

PSCs are available as commercially mature cell lines. In contrast, adult stem cells and primary cells are generally obtained from biopsies or surgical resection samples. These samples, which have undergone enzymatic digestion to degrade the ECM, can be further incubated with collagenase, elastase, or dispase to generate single-cell suspensions, depending on the tissue or organ of origin [51–53] (Fig. 2 a). Subsequently, target cells are isolated for cultivation via magnetically activated cell sorting or fluorescence-activated cell sorting based on specific biomarkers or physical parameters [16, 54, 55].

#### 3D ECM construction

Upon preparation of the cells, they would be inoculated into a 3D ECM (Fig. 2 b). Depending on the controllability of positioning, regulation, and spatial resolution, this process can be classified into two approaches: matrixgel embedding and bioprinting [25, 56]. According to the heterogeneity and composition, the matrices can be categorised into matrixgel and hydrogels, with the latter further subdivided into natural and synthetic types [57–59].

##### Matrigel

Matrigel, the most widely used ECM material, is derived from the Engelbreth-Holm-Swarm tumor, a unique source of ECM that was fortuitously discovered in mice over four decades ago [60]. This matrix primarily comprises laminin, collagen type IV, entactin, nidogen, perlecan, and various growth factors. Matrigel not only supports adhesion across a broad spectrum of cell types, including stem cells, epithelial cells, endothelial cells, and tumor cells, but also provides essential signals that promote cellular differentiation [61]. When initially inoculating, cells were suspended in pre-cooled matrigel and subsequently introduced into the culture dish. As the temperature increased, the matrigel gelled and expanded to form a scaffold, then covered with medium containing growth factors, promoting cell self-organization and organoid formation within the 3D microenvironment. In practical applications, ensuring the consistency of the gel dome remains challenging [62]. Even with automated processes that guarantee uniformity, variations in organoid size may still occur due to paracrine effects or differences in permeability efficiency [63]. While Matrigel is currently the most widely utilized matrix for organoid culture, it presents several limitations: high cost, undefined composition, significant batch-to-batch variability, and susceptibility to contamination from external sources. These factors collectively hinder standardization and scalability [64].

##### Hydrogels

Hydrogels constitute a class of 3D polymer networks, exhibiting tunable physicochemical properties. Besides in situ gelation akin to matrix adhesion, various techniques can be employed to construct ECMs, including freeze-drying, electrospinning, micropatterning, microfluidics, and bioprinting [65, 66]. The characteristics of these ECMs are influenced by the material composition, concentration, cross-linking methodology, density, and preparation technique. Hydrogels can be categorized into two main types based on their origin: natural hydrogels and synthetic hydrogels. Natural hydrogels, such as collagen, gelatin, hyaluronic acid, and chitosan, exhibit excellent biocompatibility and biodegradability but possess limited stability and mechanical strength. In contrast, synthetic hydrogels, including polyethylene glycol derivatives, polycaprolactone, polyvinyl alcohol, etc., demonstrate superior mechanical properties following chemical cross-linking [67]. Although their biological activity and biocompatibility may not match those of natural hydrogels, they can endure significant mechanical stress.

In recent years, the utilization of acellular matrices for organoid construction has gained significant traction. Acellular matrices encompass all tissue components except for cellular elements, removing antigenic components that induce immune rejection while preserving the original spatial structure of ECMs along with certain growth factors [68]. It has demonstrated significant differentiation promotion, structural reproducibility, and functional efficacy in the construction of retinal organoids [69], liver organoids containing bile ducts [70], and brown adipose organoids for transplantation [71]. Given the modelling velocity, this approach holds considerable potential for infection research, drug screening, and clinical applications.

##### 3D bioprinting

Bioprinting represents an advanced application of 3D printing technology within the biomedicine field. This innovative technique facilitates the precise deposition of cells, growth factors, and biomaterials into complex 3D constructs [56]. By sequentially layering materials within a predefined support structure, bioprinting enables the creation of intricate biological architectures. Depending on the specific printing methodologies employed, bioprinting techniques can be categorized into inkjet, extrusion, and laser-assisted approaches. Each method exhibits distinct advantages and limitations concerning cell viability, tissue resolution, and economic considerations. Owing to its high-throughput potential and the capability to replicate the complex air-cell interface of the respiratory system, this technology demonstrates significant promise for advancing the study of viral infections. Deniz et al. successfully reconstructed the nasal mucosal epithelium by depositing primary human nasal epithelial progenitor cells onto transwell inserts using droplet-based high-throughput bioprinting, resulting in highly differentiated and tightly connected epithelial cells [72]. In comparison to manual seeding methods, this approach demonstrated increased sensitivity to active infection following influenza virus exposure. In comparison with traditionally cultured 2D cells, the virus propagation kinetics within the 3D microenvironment exhibited significant differences [73]. Furthermore, FDA-approved drugs demonstrated varying degrees of antiviral activity when applied to infected organoids as opposed to 2D cultured cells.

#### Soluble inducer

The ECM not only transduces adhesion signals but also binds soluble growth factors, regulating their distribution, activation, and presentation to cells through an exquisitely coordinated spatiotemporal process [74]. When stem cells are encapsulated within 3D scaffolds, growth factors pre-incorporated into hydrogels or bioinks provide critical signalling cues for cellular adhesion, proliferation, and early differentiation. Subsequent supplementation of specific growth factors through culture medium replacement is implemented according to the developmental timeline of organoids [75]. However, the induction of complex multi-lineage architectures from iPSCs demands sophisticated regulatory strategies, including sustained release mechanisms, conditional activation, concentration gradient engineering, and even autonomous cellular secretion control [34]. This necessitates the integration of advanced tissue engineering technologies to achieve precise, dynamic, and standardised regulation. One established approach involves encapsulating growth factors within nanoparticles to enable sustained and tunable release kinetics [76, 77]. Furthermore, microfluidic systems have emerged as powerful tools for reconstructing stem cell niches with precise spatiotemporal control over mechanochemical microenvironments. These systems enable the fine-tuning of environmental parameters through targeted fluid flow patterns and gradients of gaseous or molecular factors [78, 79]. Notably, microfluidic neural tube devices have been successfully employed to govern growth factor concentration gradients and achieve high-fidelity recapitulation of corresponding anatomical structures [80].

#### Physical controls

The growth and differentiation of stem cells are regulated not only by biochemical signals but also by physical stimuli, including temperature, fluid dynamics, tissue geometry, matrix stiffness, and gas environment. Furthermore, these regulatory mechanisms are essential for the simulation of the unique complex structures of various organs, thereby promoting their functional maturation and long-term culture [81].

A cell incubator is typically maintained at 37 °C to replicate the physiological temperature at which growth factors activate signaling pathways, thereby promoting the differentiation and maturation of organoids. Elevated temperatures may induce heat stress, potentially leading to stem cell damage [82], whereas lower temperatures are utilized for organoid preservation and transportation. Studies investigating seasonal coronavirus (229E, OC43, NL63) infections in differentiated human respiratory organoids at 33 °C (simulating the cooler environment of the upper respiratory tract) and 37 °C (core body temperature) have demonstrated that lower temperatures significantly enhance viral replication efficiency and elicit unique host transcriptome responses, including the activation of inflammatory pathways [83].

Static organoid cultures typically depend solely on routine medium exchanges to enhance nutrition and remove metabolic waste. Microfluidic systems and rotary bioreactors offer a more sophisticated fluidic environment, maintaining consistent hydrostatic pressure and shear stress, among other factors, to activate mechanosensitive signaling pathways such as YAP/TAZ. This promotes the differentiation of stem cells into specific lineages and organoid vascularization [84, 85]. It has been demonstrated that in the microphysiological chip system for polycystic kidney disease, organoids subjected to fluid shear stress expand cysts via an absorption pathway rather than a secretion pathway [86]. Ma et al. developed a microfluidic chip capable of generating a constant shear force, which was shown to increase VEGF release and induce greater tumor cell damage and necrosis [87]. These findings indicate that enhancing the fluidic environment of organoids using tissue engineering techniques not only supports their normal metabolic functions but also elucidates the influence of previously overlooked hydrodynamic parameters on disease progression and emerging therapeutic strategies.

Controlling the morphology of organoids through the construction of an ECM is a critical determinant influencing their differentiation, growth, and metabolic efficiency. Modulating the size, shape, structure, and cellular arrangement of organoids can significantly impact these processes [88]. Specifically, the size of organoids directly affects nutrient and oxygen concentration as well as the diffusion of metabolic waste [89]. Large organoids require perfusion-based culture systems, porous hydrogels, or vascularized structures to enhance nutrient penetration [90, 91]. Shape, in turn, influences cell behavior via mechanical stress transmission and spatial signaling gradients. For instance, globular organoids, such as brain organoids, spontaneously develop neuroepithelial layered structures [92], while tubular organoids like renal tubules promote functionalization through apical-basal polarity [93]. Branching bile duct organoids rely on the JAG1/NOTCH2 signaling pathway to compensate for the lack of pancreatotropin and somatostatin receptor activity observed in cystic organoids [94]. Moreover, structures that mimic the spatial complexity of in vivo tissues play a pivotal role in determining functional maturity. Stent-guided intestinal organoids forming tubular structures with unobstructed lumens and crypt-villus-like architectures can be perfused to continuously remove dead cells, thereby extending tissue viability by several weeks [95]. These structures also enable microbial colonization to simulate host-microbe interactions. Maurat et al. utilized alginate gel tubular scaffolds to induce epithelial cells to self-organize into tubular structures, creating a bronchial model capable of recapitulating distal airway characteristics [96].

ALI culture represents an advanced technique in tissue engineering, wherein cells are induced to establish apical-basal polarity by exposing the apical surface of organoids to gas while maintaining the basal side immersed in culture medium. This method facilitates self-organization processes that promote the formation of layered structures, such as hair follicle development in skin organoids [97] and ciliary-mucus layer formation in respiratory epithelial organoids [98]. Compared to conventional complete immersion culture, ALI enhances oxygen and nutrient diffusion, thereby reducing hypoxia-induced cell necrosis within organoids and more closely mimicking the physiological interactions between organs and their external environment. Consequently, this approach improves predictive accuracy in drug permeability testing and pathogen infection modelling [49]. ALI technology has been extensively applied in the development of skin, respiratory tract, and intestinal organoids, offering a robust in vitro platform that closely replicates the in vivo microenvironment for applications in disease modelling and regenerative medicine research.

#### Immune organoid and those co-cultured with immune cells

Organs and tissues within the body are subject to the influence of various physical and chemical factors, as well as being regulated by the nervous and immune systems. The organoid immunoco-culture system markedly enhances the maturity and functionalization of organoids through the integration of immune cells and tissue-specific cells, thereby providing a highly biomimetic platform for investigating host–pathogen interactions and immune regulation mechanisms [99].

For instance, Kang et al. developed a co-culture system combining PSCs-derived alveolar epithelial organoids and induced macrophages, identifying alveolar type 2-like cells producing GM-CSF and macrophage-like cells exhibiting core immune functions through single-cell RNA sequencing and functional analyses [100]. Under conditions of injury or infection, these assembloids replicate key aspects of human respiratory defence, wherein the macrophage-like cells effectively eliminate damaged cells and internalise oxidised lipids. Seo et al. developed alveolar organoids incorporating functional macrophages, which offer significant advantages for the investigation of infectious diseases, highlighting the importance of immune cell presentation in organoids for modelling inflammatory pulmonary diseases [101]. Furthermore, an organ-on-chip system established via co-culture of human pluripotent stem cell-derived cardiomyocytes and peripheral blood mononuclear cells mimics the mechanism of myocardial injury associated with COVID-19 [102]. High-throughput screening based on this model identified that the JAK inhibitor baricitinib effectively suppresses macrophage-mediated myocardial injury, and the drug has been approved for clinical treatment of COVID-19. By precisely controlling the ratio of cell populations, infection parameters, and drug interventions, such systems not only elucidate the immunopathological mechanisms triggered by viral infections but also expedite the development of antiviral drugs while providing a reliable, controllable experimental platform for personalised treatment strategies and precise immune assessments [103].

In addition, 3D tonsil organoids derived from human tonsil cells retain B cells, T cells, and stromal cell components, successfully replicating the germinal centre microenvironment and supporting somatic hypermutation and affinity maturation of antigen-specific B cells [104]. This model could modulate the expression of immune cell proteins and promote the production of specific IgG/IgA antibodies, thereby offering a novel approach for investigating individualised immune responses.

#### Organ-on-a-chip and microfluidic

The integration of organ-chip technology with a microfluidic system markedly enhances the functional maturity and physiological relevance of organoids by incorporating multi-dimensional stimulation signals, such as mechanical force, biochemical gradients, and fluid shear stress [29] (Fig. 2 c). This advancement offers a large-scale, standardised, and highly biomimetic platform for the development of viral infection models [105]. Its primary advantage resides in the precise simulation of the dynamic characteristics of the in vivo microenvironment. By incorporating an elastic membrane or pneumatic device within the chip, it can mimic organ motions such as alveolar breathing and heartbeats, thereby promoting the maturation of organoids. For instance, influenza A (H3N2) infection in human alveolar chips with periodic respiratory-like deformation was investigated. Compared to static chips, this dynamic model elicited a series of host responses, demonstrating that respiratory movement activates protective innate immune responses in both epithelial and endothelial cells, effectively inhibiting viral replication [106]. Hierarchical microfluidic channels are capable of constructing a functional vascular network and simulating the hemodynamic environment. In the blood–brain barrier (BBB) chip, endothelial cells form tight junctions under the influence of shear stress and achieve a transendothelial electrical resistance exceeding 2000 Ω·cm^2^ following co-culture with astrocytes. This model has been utilised to investigate the mechanism by which the Zika virus crosses the BBB, revealing that the virus infects nerve cells via the AXL receptor-mediated endocytic pathway [107]. Complex microenvironments, including substance exchange interfaces and nutrient/metabolite gradients, can be established via multi-channel microfluidic systems. Research has demonstrated the infection pathway of Coxsackievirus B1, which infects highly differentiated human villous intestinal epithelium through either the epithelial lining of the intestinal lumen or parallel "vascular" channels [108]. In the investigation of COVID-19, a lung chip model featuring integrated alveolar-vascular dual channels was utilised to simulate the spatiotemporal dynamics of cytokine storm following SARS-CoV-2 infection. Upon viral infection of the epithelial layer, the microfluidic system enabled real-time monitoring of the extravasation of inflammatory factors, such as IL-6 and CXCL10, through the vascular side, thereby triggering the activation of endothelial cells. Camostat, a TMPRSS2 inhibitor identified through this model, demonstrated effective blockade of viral entry, with its efficacy showing high concordance with clinical data [109]. Compared to traditional culture methods, organ-on-a-chip technology not only accelerates the elucidation of viral pathogenesis by integrating dynamic stimulation signals with high-throughput detection but also facilitates the precise evaluation of antiviral drugs and vaccines, thereby providing an in vitro model that more closely mimics human physiology for the study of infectious diseases.

On the other hand, the integration of microfluidic systems with organoid high-throughput detection platforms has significantly enhanced the efficiency and reproducibility of organoid research by enabling standardisation, automation, and parallel processing. This advancement provides a powerful and innovative tool for virology research and drug development [110]. Microfluidic chips enable the realisation of batch culture and simultaneous detection of organoids via multi-channel designs. For instance, in anti-SARS-CoV-2 drug screening, lung-on-a-chip arrays equipped with integrated optical sensors can concurrently evaluate the effects of hundreds of compounds on viral replication (as detected by fluorescently labelled viral RNA) and cytotoxicity (as assessed by LDH release). This approach significantly reduces the screening cycle from weeks to days [111]. High-throughput platforms based on alveolar microarray technology have been utilised to assess the efficacy and cross-barrier permeability of drugs such as remdesivir and baritinib. Their predictive outcomes exhibit a high degree of consistency with clinical trial data [111]. By automating the addition of various vaccine candidate antigens (e.g., spike proteins encoded by mRNA vaccines) in tonsil organoid chip arrays, the processes of B cell affinity maturation and antibody neutralisation titers can be concurrently monitored, thereby accelerating vaccine optimisation [104]. These platforms integrate organoid culture, viral infection, drug intervention, and phenotypic analysis within the closed-loop framework of "design-build-test," which not only facilitates the transition of virology research from static to dynamic models and from single-organ to multi-organ systems but also provides efficient and reliable in vitro models for infectious disease prevention, control, and precision medicine applications.

### Identification, morphological and functional validation

**Fig. 3:**
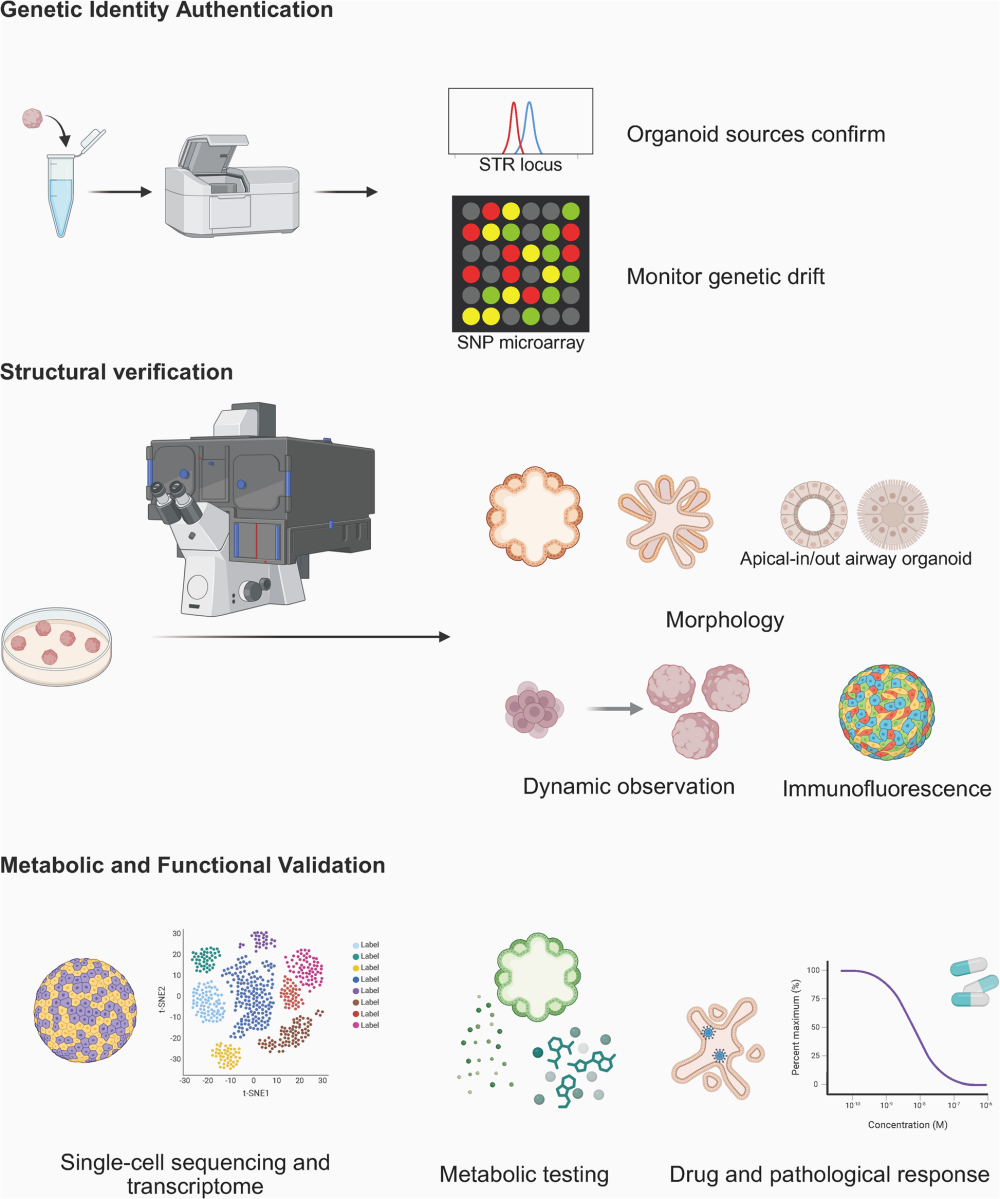
Identification, morphological and functional validation of organoids Genetic identity authentication: STR sequences were utilised to authenticate the origin of organoids and prevent cross-contamination, while SNP analysis was employed to detect genetic drift resulting from long-term passaging. Structural verification: The morphology, composition, and dynamic changes of organoids were systematically observed under the microscope, and cellular composition was analysed using immunofluorescence staining. Metabolic and Functional Validation: Organoids should preserve the gene expression profiles (as determined by transcriptomic analysis), secretory and metabolic functions characteristic of the originals, and exhibit comparable responses to stimuli such as viruses and drugs. (Created in https://BioRender.com)

The successful construction of organoids must meet specific criteria to meet research and development requirements while preserving structural and functional fidelity to the original tissues (Fig. 3). In the absence of a unified standard for organoid identification, authentication of these in vitro cell culture systems could be guided by criteria analogous to those for cell line authentication.

#### Genetic identity authentication

Short tandem repeat(STR) sequences provide a robust and reliable method for confirming the uniqueness of organoid sources and preventing cross-contamination [112], thereby facilitating comparisons with external databases. Moreover, single-nucleotide polymorphism(SNP) detection provides high sensitivity, which is ideal for monitoring genetic drift during long-term passages [113]. Nevertheless, 3D organoids may exhibit variability in STR peak areas due to cellular heterogeneity, thus requiring the application of microdissection techniques to enrich target cell populations [114].

#### Structural verification

The morphological examination of organoids is a critical step in validating their structural and functional integrity (Table 1). Organoids capable of reproducing cell polarity exhibit markedly superior performance compared to those with random cellular arrangements, particularly in terms of functionality, metabolism, and practical applications. When combined with artificial intelligence, this process enables automated, efficient, quantitative, and precise measurement and analysis [119].

**Table 1:** The structural fidelity and functional complexity of organoids of respiratory viral infections

Organoid type	Structure	Function	Sensitivity and accuracy advantage
Airway Organoid [115]	Pseudostratified ciliated columnar epithelium: oriented and tightly packed with predominantly ciliated cells	Mucociliary clearance function Cilia motility Barrier protection function	Characterisation of active viral infection
Brain Organoid [116, 117]	Cortical laminar organisation Blood–brain barrier	Neuronal electrical activity Glutamatergic receptor activity Barrier protection function	Multiple organ infection Mechanisms of virus invasion into the nervous system
Intestinal Organoid [16]	Crypt-villus polarity	Intestinal epithelial absorption, mucus secretion and stem cell homeostasis	Cross-species infection Mucosal immune response
Liver Organoid [118]	Hepatic lobule-like arrangement Bile duct network	Enhanced activity of CYP450 enzymes Metabolic capacity of bile acids	Accurate assessment of drug-induced liver toxicity Pathological mechanism of non-alcoholic fatty liver disease
Kidney Organoid [93]	Glomerular- and tubular-like compartments Renal tubules' polarised epithelial structure	Glomerular filtration barrier Substance transport in renal tubules	Receptor blocking infection

Initially, basic morphological evaluation systematically assesses macroscopic characteristics using light microscopy, focusing on parameters such as the diameter of intestinal organoids, cavity integrity [120], and the branching complexity of pulmonary organoids. Confocal microscopy's imaging capabilities facilitate detailed spatial arrangement analysis of cells, while immunofluorescence labelling confirms the polarity distribution of epithelial cells [42, 121]. Electron microscopy reveals ultrastructural features, such as microvillus density in intestinal organoids or synaptic maturity in brain organoids [122, 123]. Throughout the entire culture cycle, continuous dynamic morphological monitoring is essential, particularly during the initial construction phase (24–72 h) to track cell aggregation patterns. For instance, intestinal organoids are anticipated to develop into a closed spherical structure with a diameter of 50–100 μm, and organ-specific compartmentalization should be confirmed during the maturation phase (7–14 days) [16]; brain organoids must exhibit a six-layered cortical architecture [116]; while pulmonary organoids should form structures resembling terminal bronchioles [52].

Furthermore, the histological analysis of organoids necessitates staining procedures. Hematoxylin and Eosin (H&E) staining is employed to validate the structural resemblance between organoids and their original tissues, exemplified by the crypt-villus architecture in intestinal organoids [124]. Additionally, specialized staining techniques are utilized to assess functional characteristics: Periodic Acid-Schiff staining quantifies mucus secretion from goblet cells [125], while Masson's trichrome staining identifies interstitial collagen deposition, as observed in liver fibrosis models [126]. Molecular markers facilitate the precise quantification of cell composition. Immunofluorescence and histochemistry can detect key markers of specific cell subsets. The deviation in their proportions from the original tissue should not exceed 10%. Flow cytometry enables the quantification of dynamic changes in cell subset proportions, such as Alb + hepatocytes, CK19 + cholangiocytes, and CD31 + endothelial cells in liver organoids [127]. Spatial omics technology can map the spatial distribution of cell types and validate the regional consistency between organoids and original tissues [128].

Integrating the aforementioned strategies, a verification system was proposed. In the initial phase, a comprehensive morphological and compositional analysis of the entire sample was conducted. Thereafter, key indicators such as the H&E structure score and the proportion of the top three markers were re-evaluated every five generations to ensure experimental reproducibility.

#### Metabolic and functional validation

The functional validation of organoids necessitates a comprehensive assessment of their secretory, metabolic, electrophysiological, and molecular responsiveness properties to ensure faithful replication of the original organ's physiological characteristics. Stable metabolic and functional integrity guarantees accuracy and comparability in virological research applications.

Multi-group strategies offer a comprehensive molecular foundation for elucidating the gene regulatory mechanisms underlying organoid metabolic functions, including quantitative PCR quantification of key functional genes, single-cell RNA sequencing (scRNA-seq) analysis to assess cellular heterogeneity, and epigenetic analyses [129]. Through scRNA-seq, Jitsuke et al. demonstrated that SARS-CoV-2-infected iPSC-derived kidney organoids exhibited cellular damage and dedifferentiation while activating pro-fibrotic signalling pathways [130]. Targeted metabolic profiling is the cornerstone of the technical framework for validating organoid metabolic homeostasis. Metabolic markers can be employed to assess the absorptive efficiency, secretory function, and enzymatic activity in organoids such as the liver and intestine [131–134]. Duan et al. demonstrated that metabolic profiling of PSCs-derived airway organoids revealed GW6471-mediated suppression of HIF1α-driven glycolysis to block SARS-CoV-2 infection, with parallel inhibition by fatty acid biosynthesis inhibitors, defining the HIF1α-glycolysis axis as a critical metabolic checkpoint for viral entry [135]. The evaluation of pharmacological and pathological metabolic responses highlights the critical role of organoids in translational medicine. By integrating cell viability and toxicity assays, viral infection/replication assessments, and analyses of metabolic pathway specificity, the incorporation of organoid-based drug metabolism kinetics with pathological metabolic profiling can significantly improve the clinical translational efficiency of respiratory virus research [136]. Elucidation of electrophysiological and mechanometabolic activities provides insight into the advanced functional features of specific organoids. Ion imaging technology enables the recording of spontaneous calcium oscillation frequencies in neuronal organoids, with abnormal rhythms indicating potential developmental defects [137]. Patch-clamp techniques or photoelectrochemical imaging can quantify electrophysiological properties and measure action potential duration and sodium current density in cardiac organoids [138]. Moreover, the pulse intensity and rhythm of cardiac organoids can be directly recorded for video analysis [139].

Following rigorous validation of organoid identity and functional maturity, these physiomimetic models serve as robust platforms that bridge benchside research with clinical translation, thereby facilitating precision-based drug screening and personalised disease modelling.

## Application of organoid model in virus research

Respiratory virus research has historically relied heavily on animal models, yet significant limitations persist, including interspecies differences, ethical concerns, and discrepancies from human physiological traits. Therefore, organoids are gaining prominence in virology due to their personalised origins, highly consistent physiological characteristics, and robust manipulability. These models offer crucial tools for infection modelling, mechanistic studies, antiviral drug screening, and vaccine development.

### Establishment and mechanism study of comparable infection models

Compared to the conventional 2D cell culture model, organoids are capable of mimicking the intricate microenvironment of human organs and offer a more accurate representation for studying the dynamic processes of viral infections. Organoids serve as critical tools in investigating viral invasion and replication, tissue-specific infections, virus-host interactions, viral transmission, and cross-species infections.

Combined with gene editing and high-throughput screening, organoids serve as a robust platform for the rapid identification of host genes associated with viral invasion and replication. Joep Beumer et al. established organoid-based knockout biobanks to validate ACE2, the receptor for SARS-CoV and SARS-CoV-2, as well as DPP4, the receptor for Middle East respiratory syndrome coronavirus(MERS-CoV) [140]. The acceleration of therapeutic intervention development hinges on the identification of host genes implicated in viral pathogenesis. Han et al. utilised lung and colon organoids to demonstrate significant anti-infective effects of inhibitors targeting the SARS-CoV-2 receptor, ACE2 [136]. Organoids of host animals have been employed to explore the potential of cross-species transmission of viruses. Bat intestinal organoids have demonstrated that SARS-CoV-2 can infect bat intestinal cells, while the virus exhibits robust replication in human intestinal organoids, suggesting that the human intestine may serve as a transmission route for SARS-CoV-2 [141]. Advanced organoid models, such as organ-on-a-chip systems, recapitulate multicellular interactions and tissue barrier functions (e.g., alveolar and blood–brain barriers), enabling comprehensive evaluations of the antiviral, anti-inflammatory, and tissue-protective effects of drugs [142].

In view of the wide applicability and sensitivity, organoids enable systematic comparison of infection kinetics, immune escape mechanisms, and tissue tropism differences among various respiratory viruses, including influenza virus, RSV, and SARS-CoV-2. Additionally, drug candidates targeting conserved viral or host factors (e.g., viral RNA-dependent RNA polymerase, host proteases) as well as universal vaccine candidates can be evaluated in parallel. For instance, Remdesivir has been utilised to concurrently validate its inhibitory effects against MERS-CoV, SARS-CoV-2, and parainfluenza viruses within a single model, thereby expediting the development cycle [143]. Furthermore, ferritin nanoparticle-based pan-coronavirus vaccines can be tested for their protective efficacy against both alpha and beta coronaviruses in challenge experiments, accelerating preclinical validation of broad-spectrum vaccines [144].

Compared to the relatively mature evaluation system of animal models, the current organoid research exhibits significant gaps in process standardisation, data comparability, and infection assessment criteria. The utilisation of uniform cell sources, standardised medium formulations, and culture conditions in accordance with established guidelines is crucial for ensuring the comparability of study data. Mature stem cell lines or organoid banks can serve as valuable resources for virological research. When cells derived from individualised sources are utilised for culture, comprehensive genetic background information and verification data must be provided to ensure reliability and reproducibility. Referring to well-established organoid culture guidelines facilitates the establishment of a unified standard for constructing respiratory organoids, thereby minimising operational discrepancies between laboratories [145, 146]. A thorough transcriptomic and morphological assessment of various kidney organoid culture protocols has demonstrated that kidney organoids derived from different human iPSC sources exhibit robustness, reproducibility, and comparability. The primary source of variation lies in technical parameter differences; therefore, it is essential to control as many technical variables as possible during the culturing process [147]. Even in central nervous system organoids, which exhibit high cellular diversity, reproducible developmental induction can occur independently of an embryonic background. Under fixed culture conditions, organoids originating from diverse stem cell sources and growth environments consistently form similar structures and terminal cell compositions [92].

### High-throughput screening and drug development platform

By replicating the in vivo respiratory tract interface, organoids serve as a robust platform for high-throughput drug screening. When integrated with automation technologies, the precision of system predictions is enhanced, and the drug discovery cycle is substantially accelerated. In airway organoids, the EC_50_ values of drugs were observed to be more than fivefold lower compared to conventional cell lines, such as Vero or Calu-3 cells, thereby demonstrating superior predictive accuracy. Furthermore, organoids enable the assessment of drug toxicity on host cells, diminishing reliance on animal models while improving the reliability of preclinical predictions. This highly biomimetic model not only expedites drug target validation but also offers critical insights for refining drug design (e.g., enhancing specificity and minimising adverse effects) [110]. Organoids provide a dynamic analytical framework for multi-drug combination studies. By mimicking viral infection processes, researchers can systematically evaluate the synergistic or antagonistic interactions of various drug combinations (e.g., antiviral agents and immunomodulators), thus optimising therapeutic strategies [148]. Additionally, organoids can simulate the evolution of viral drug resistance through prolonged infection modelling, enabling the identification of drug combinations that overcome resistance and offering innovative approaches for the clinical management of drug-resistant strains [149].

Organoids serve two critical functions in vaccine development. First, they can elucidate the interaction mechanisms between viruses and hosts, thereby providing critical targets for vaccine antigen design. Moreover, organoids can be utilized to directly evaluate vaccine-induced immune responses. For instance, the human tonsil organoid model has been employed to track the differentiation and dynamics of adaptive immune responses triggered by influenza vaccines, exploring how antigen-specific B and T cells are activated and contribute to mucosal immune responses [150]. Additionally, respiratory organoids can replicate the mucosal immune interface, offering a research platform for developing mucosal immune strategies, such as nasal spray vaccines. These strategies can more effectively block virus transmission by activating local immune responses [151].

### Personalisation and precision medicine

Individual responses to respiratory viruses exhibit inherent variability, influenced by differences in receptor expression and immune-related genetic factors. For instance, the expression levels of the ACE2 receptor are closely associated with the risk and severity of SARS-CoV-2 infection [152]. The HLA genotype modulates the efficiency of antigen presentation to the immune system and is recognised by specific cytotoxic T lymphocytes during influenza virus infection [153]. Furthermore, individual variations in baseline immune capacity, preexisting conditions, and immunological memory contribute to differential outcomes. For example, older adults and immunocompromised individuals are at higher risk of developing severe disease upon exposure to SARS-CoV-2 [154]. Patients with asthma or chronic obstructive pulmonary disease may experience exacerbated airway hyperresponsiveness and reduced lung function following viral infection [155]. Conversely, cross-reactive immunity acquired through prior infection or vaccination may mitigate symptom severity upon re-exposure to related viruses [156]. Organoids derived from patient-specific iPSCs or biopsy tissues retain patient-specific characteristics, including genetic variants and epigenetic modifications [157]. This approach enables precise modelling of individual susceptibility to viral infections. By screening antiviral agents or immunomodulatory compounds using these personalised organoids, clinicians can enhance treatment efficacy, minimise adverse effects, and optimise resource utilisation.

### Organoids in the age of artificial intelligence

In the era of artificial intelligence (AI), organoids have been developed with greater precision and efficiency within the aforementioned application fields. By integrating with machine learning and deep learning algorithms, they offer more efficient and intelligent approaches to analysing viral mechanisms, developing drugs, and advancing precision medicine. Organoids equipped with AI interfaces not only enable automated experiments and accurate predictions but also facilitate the integration of data from single organs to multi-organ systems, thereby realising the concept of a "human on chips" [158]. This advancement can expedite the research and development of antiviral drugs and vaccines while enhancing the rapid response capability to emerging infectious diseases. As computational power improves, algorithms are optimised, and interdisciplinary collaborations deepen, AI-assisted organoid models are anticipated to become a pivotal hub connecting basic research, drug development, and clinical applications, providing innovative solutions to address global public health challenges.

Massive multi-dimensional datasets, such as high-resolution imaging, gene expression profiling, and metabolic dynamics generated by organoids, can be effectively analysed using advanced AI algorithms. Deep learning-based image analysis technologies enable the automatic quantification of virus-infected areas, cytopathic effects, and drug repair efficacy in organoids, thereby significantly enhancing the efficiency of data extraction. By integrating transcriptomic, proteomic, and metabolomic data, AI has facilitated researchers' understanding of the molecular mechanisms underlying cardiac development [159] and drug discovery on tumor organoid platforms [160], potentially revealing critical pathways of viral infection and predicting potential drug targets. Machine learning not only constructs dynamic models of virus-host interactions to simulate viral replication cycles, immune escape mechanisms, and other processes, providing a theoretical foundation for antiviral strategies, but also develops epidemiological models to predict viral transmission trends and the impact of mutations. Organotypic cultures of human cortical brain explants (OPAB) have been explored as a preclinical platform for AI-driven antiviral research. A machine learning framework was developed to predict OPAB infection status with high confidence and validated through antiviral treatment experiments [161].

AI not only supports existing organoid-based drug development platforms but also subtly transforms the paradigm of antiviral drug development. Virtual screening approaches, such as those based on deep learning or directed information transfer neural networks, have demonstrated high accuracy and specificity in predicting and validating anti-SARS-CoV-2 drugs [162, 163]. High-precision structure-based virtual screening can predict drug binding poses and affinities. Some studies have successfully screened billions of compounds within seven days [164]. Such methods are anticipated to predict the binding capacity of compounds to viral targets, prioritise the selection of high-potential molecules or drug combinations, and subsequently validate these predictions through organoid experiments, thereby significantly reducing experimental costs.

## Organoid models for respiratory virus infection

**Fig. 4:**
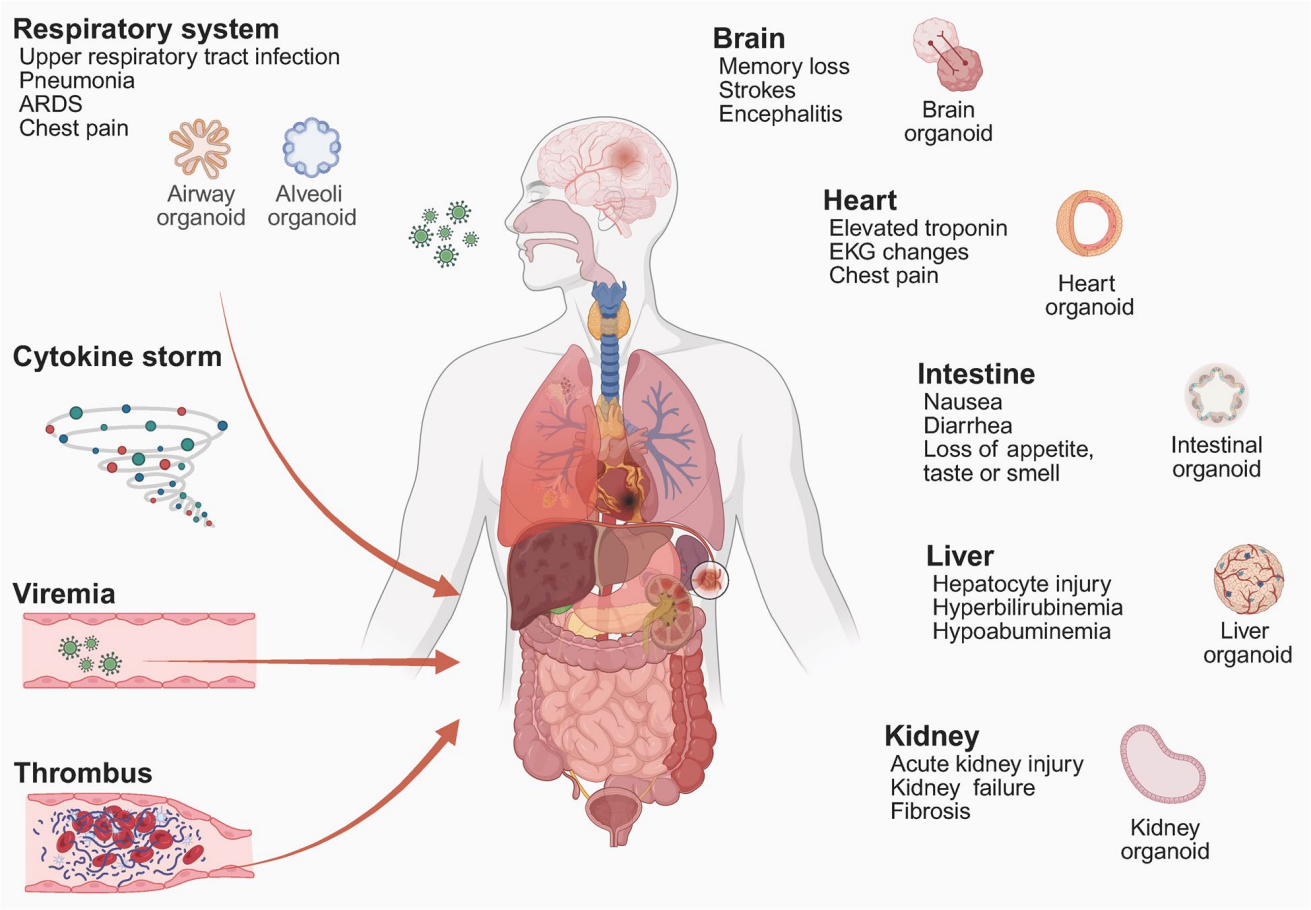
Severe respiratory viral infections may result in multi-organ failure Respiratory viral infections originate in the respiratory tract, where viral replication induces localised inflammation and alveolar injury. Viral particles or components may spread hematogenously or through neural routes, resulting in extrapulmonary manifestations. In the brain, neuroinvasion can lead to encephalitis, neuroinflammation, or blood–brain barrier compromise. Gastrointestinal involvement stems from viral entry via ACE2 receptors. As for the liver and kidneys, they may sustain damage due to direct viral invasion, cytokine storms, or immune dysregulation. Systemic inflammation and endothelial dysfunction further contribute to multi-organ dysfunction. (Created in https://BioRender.com)

Respiratory viral infections continue to represent a significant global public health challenge. Infection with SARS-CoV-2 can lead to a spectrum of complications, including psychiatric disorders, cognitive and physical impairments, venous thrombosis, myocarditis, acute cerebrovascular disease, heart failure, and liver injury (Fig. 4). Over the past few decades, seasonal outbreaks of influenza viruses have resulted in millions of severe cases and fatalities worldwide [165]. The current absence of rapid and reliable in vitro models for predicting influenza virus infectivity has significantly impeded efforts in influenza prevention and control. Influenza A virus typically induces acute febrile respiratory symptoms following entry into the respiratory tract [166]. Given its short incubation period, this virus may pose an elevated risk, potentially leading to acute pneumonia in individuals with chronic conditions. However, due to the antigenic diversity of the virus, investigations into infection and pathogenesis must be conducted using specific antigen types [167]. RSV predominantly affects infants and young children, causing lower respiratory tract infections such as acute pneumonia, including inflammation and constriction of the small airways [168].

The virological characteristics of these respiratory viruses change rapidly over time due to their high mutation frequency. The growing demand for fundamental and translational research necessitates the development of in vitro and in vivo models that are low-cost, highly operable, high-throughput, and highly simulative. Prior to the emergence of SARS-CoV-2, organoids were predominantly used to study human biology and development, with limited application in infectious diseases. In recent years, the ongoing SARS-CoV-2 pandemic has significantly heightened the focus on organoids, which are now widely employed in both basic research and the discovery of countermeasures against SARS-CoV-2. Owing to advancements in 3D culture techniques and the inherent advantages of organoids, they have emerged as optimal models for investigating viral invasion and replication, tissue tropism, and host-virus interactions [41, 169].

### Respiratory organoids

**Fig. 5:**
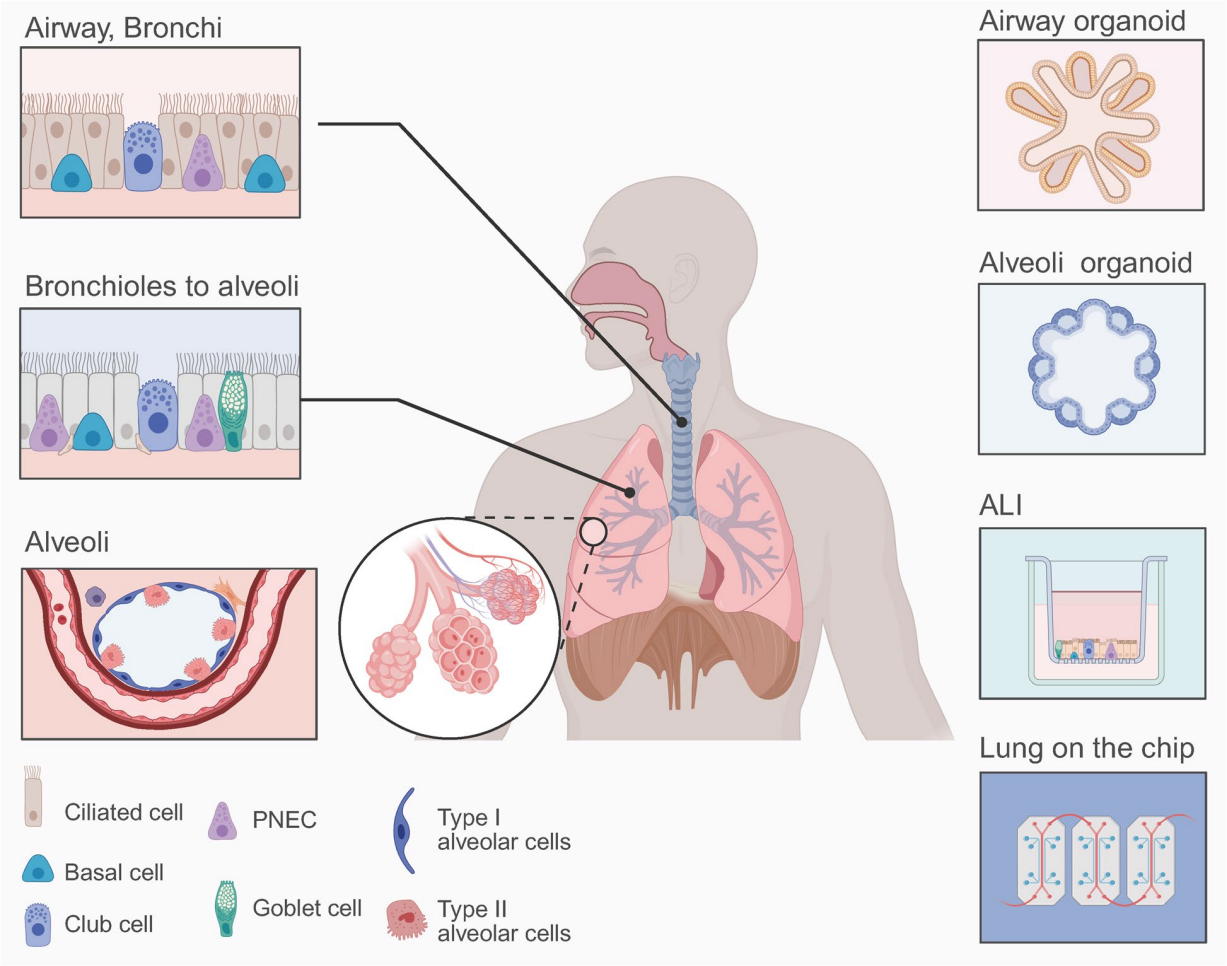
Respiratory system structures, cellular components, and corresponding organoids The respiratory tract, extending from the middle and posterior regions of the nasal cavity to the bronchi at all levels, is lined with pseudostratified ciliated columnar epithelium, comprising ciliated cells, goblet cells, basal cells, club cells, etc. Organoids that mimic this specific region are termed airway organoids. These epithelial cells can also be derived from primary cells or induced differentiation of PSCs, form a monolayer structure on transwell inserts and can be maintained under ALI culture conditions. Alveolar organoids replicate the composition of alveolar epithelium. However, to achieve gas–liquid exchange or coordination with the airway, it is essential to integrate them with microfluidic systems or serial chip systems. (Created in https://BioRender.com)

The respiratory system comprises the upper airways, which include the nose, pharynx, and larynx, and the lower airways, which encompass the trachea and lungs. The lung serves as the central organ of the respiratory system and is the primary target for respiratory viruses. Its intricate histological architecture facilitates efficient gas exchange and immune defence mechanisms. This includes the conducting portion, composed of the trachea, bronchi, and bronchioles, and the respiratory portion, consisting of respiratory bronchioles, alveolar ducts, and alveolar sacs. Additionally, the interstitial component is rich in blood vessels, lymphatic vessels, nerves, and immune cells. Consequently, based on simulated anatomical structures, respiratory system-related physiological organoids can be categorised into nasopharyngeal organoids, bronchial organoids, alveolar organoids, and composite lung organoids (Fig. 5). Among these, airway organoids refer to organoids that encompass the entire respiratory tract, including the nasopharynx, trachea, bronchi, and bronchioles [40, 170].

The upper respiratory tract serves as the entry point and primary site of infection for respiratory viruses, making nasopharyngeal or airway organoids uniquely valuable for studying initial viral infections. Based on their cellular origin, airway organoids can be further classified into PSC-derived airway organoids and ASC-derived airway organoids. The former is particularly suited for developmental biology and genetic research, while the latter more closely mimics adult physiology and is ideal for rapid translational applications. Given that PSC-derived organoids can replicate the infection mechanism of viruses on immature airways, this model is applicable for investigating the severe exacerbation of RSV in infants [171]. ASCs-derived airway organoids can rapidly and precisely simulate the dynamics of proximal respiratory tract infections. By utilising primary cultures of nasal epithelial organoids, researchers demonstrated that the SARS-CoV-2 virus enters the cell body by binding to motile cilia via ACE2 receptors [151]. Subsequent investigations employed nasal organoids constructed from nasal epithelial cells to infect SARS-CoV-2, thereby rediscovering the differences and infectivity of various strains [170]. In comparison with other airway organoids, bronchial organoids more closely mimic the microenvironment of the bronchi, particularly the bronchi and bronchioles. Despite their branched structure, bronchial organoids exhibit greater branching complexity and contain a higher abundance of Club cells (which secrete antiproteases), neuroendocrine cells (involved in signal regulation), and basal stem cells (responsible for epithelial regeneration). Their cellular composition is thus more intricate [172]. In human bronchial epithelial cell line-derived bronchial organoids, SARS-CoV-2 induces paracrine senescence through cytokines secreted by infected cells, a phenomenon that cannot be adequately explored in conventional plate culture models lacking cellular heterogeneity [173]. The apical-out airway organoids exhibit a higher susceptibility to viral infections, making them an attractive model for scholars. Airway organoids cultured in suspension without ECM demonstrate distinct responses to antiviral treatments and enable the study of host–pathogen interactions with greater throughput compared to others [174]. Following SARS-CoV-2 infection, these organoids can sustain efficient and multi-cycle viral replication, accurately recapitulating the enhanced infectivity and replication fitness of Omicron variants BA.5 and B.1.1.529 relative to the ancestral virus [175].

The lung tissues, encompassing respiratory bronchioles, alveolar ducts, alveolar sacs, and alveoli, serve as the central regions for gas exchange and constitute the respiratory portion of the lung. These areas are sometimes referred to as distal lung tissue, although this term may also include terminal bronchioles that lack gas-exchange functions. The alveoli consist of two primary cell types: type I alveolar cells (AT1) and AT2. AT1 facilitates gas exchange with the surrounding blood vessels in the alveolar wall, while AT2 secretes surfactant and supports the regeneration of AT1 cells. Naturally, AT2 are considered as primary ASCs in adult lung [38, 176]. The alveolar organoids derived from AT2 exhibited pulmonary pathological features consistent with those observed in patients infected with SARS-CoV-2 [41]. Pretreatment of these entities with a low dose of interferon markedly suppressed viral replication, suggesting that interferon may have a preventive effect against SARS-CoV-2 infection. The bipotential organoid model developed by Chiu MC et al. enables the extraction of stem cells from surgically resected human lung tissues, followed by initial expansion to form lung organoids, and subsequent induction of either proximal or distal differentiation. Airway or alveolar organoids can thus be generated within 2–3 weeks, effectively recapitulating the enhanced transmissibility and replication fitness of SARS-CoV-2 Omicron variant. Notably, AT2 were identified as the progenitor cells of alveolar organoids [177]. In addition to AT2, researchers have been actively investigating pulmonary ASC populations capable of sustaining long-term organoid culture and self-renewal [114]. Salahudeen et al. found that KRT5^+^ basal cells, which could develop lumens lined with differentiated club and ciliated cells, exhibited higher proliferation compared to AT2-derived alveolar organoids in mixed distal lung culture [42]. The distal lung organoids derived from these two stem cell populations, which possess apical-out polarity and present ACE2 on the exposed external surface, displayed distinct infection tropisms upon SARS-CoV-2 infection. Influenza virus can invade AT1 cells, disrupting the gas exchange barrier [178]. RSV replicates within this region, causing bronchiolitis [179]. Given the significant anatomical and cellular differences between the lungs of animals such as mice and humans, there is an urgent need for organoid models to elucidate the pathological mechanisms underlying these viral infections. Hans-Willem Snoeck and his team successfully achieved directed differentiation of distal lung organoids, derived from induced pluripotent stem cells, into branched airway organoids or vesicular alveolar organoids with preserved mesenchymal characteristics [180, 181]. These organoids are susceptible to infection by respiratory viruses such as RSV, parainfluenza virus, and measles virus. Notably, bronchiolitis-like symptoms were recapitulated in an RSV infection model. PSC-derived alveolar organoids cultured by another research group exhibited susceptibility to SARS-CoV-2 infection and demonstrated robust chemokine induction following SARS-CoV-2 infection, consistent with observations in patients with COVID-19 [136].

ALI culture systems have revolutionised the study of viral pathogenesis in lung organoids by preserving physiological polarity, enhancing cellular differentiation, and enabling precise modelling of host–pathogen interactions. AT2 cells derived from iPSCs and cultured at the air–liquid interface can rapidly recapitulate the initial infection of SARS-CoV-2 on the alveolar epithelial surface [182]. The transcriptomic analysis of infected cells reveals an inflammatory phenotype, and drug efficacy testing confirms the therapeutic potential of remdesivir as well as TMPRSS2 protease inhibition. Despite challenges in maintaining long-term expansion of AT2 cells, Huang et al. leveraged iPSC-derived iAT2 cells, which achieved indefinite proliferation in 3D culture before transitioning to 2D-ALI conditions on transwells [182]. This ALI platform revealed critical insights: SARS-CoV-2 infection triggered nucleocapsid protein expression in iAT2 cells, accompanied by rapid transcriptomic shifts toward inflammatory disease phenotypes, demonstrating ALI’s capacity to model dynamic host responses. Similarly, Lamers et al. utilised alveolar cells, basal cells, and rare neuroendocrine cells derived from fetal lung bud tip organoids to establish an ALI system [49]. This system not only recapitulated the susceptibility to SARS-CoV-2 infection but also delineated a therapeutic threshold of type I interferon. In general, the ALI system significantly reduces the duration of studies while preserving functional cellular structures and accurately simulating infection dynamics. This renders ALI-cultured organoids an essential tool for investigating viral tropism, immune disorders, and optimising treatment strategies in respiratory tract infections.

Composite lung organoids integrating both proximal and distal airway components have emerged as robust tools for elucidating viral pathogenesis and host responses. In the case of SARS-CoV-2, viral invasion initiates in the proximal airways, yet severe and potentially fatal disease manifestations result from the infection of AT2 cells and associated inflammatory responses in the distal lung [152]. This underscores the limitations of isolated airway models or alveolar systems in capturing comprehensive virus-host dynamics across the entire lung. The integrated airway-lung organoids (ALOs) developed by Tindle et al. can maintain both proximal and distal epithelial lineages under ALI conditions [183]. This system enables simultaneous characterisation of structural and functional aspects of the human airway. Transcriptome analysis of SARS-CoV-2-infected ALOs revealed that the proximal airway region (bronchial epithelium) sustains prolonged viral replication, whereas the distal alveolar region (AT2-to-AT1 differentiation) induces excessive immune activation, reflecting the critical hyperinflammation observed in severe COVID-19 cases.

### Brain organoids

Patients with COVID-19 may exhibit a range of neurologic complications, such as encephalitis, headache, hyposmia, and neuropsychiatric disorders [184]. Although viral particles of SARS-CoV-2 have been identified in the brain tissue or cerebrospinal fluid of some patients, the precise impact on brain function remains to be elucidated [185]. To investigate the neurotropism and pathogenic mechanisms, PSC-derived whole-brain organoids and region-specific organoids offer advanced platforms that enable high-fidelity simulation [116, 186]. Song et al. conducted a comprehensive analysis of infected brain organoids utilising single-cell RNA sequencing and identified that numerous metabolism-related neuronal genes exhibited abnormal expression patterns [185]. Furthermore, they demonstrated that neuronal infection could be efficiently inhibited by either blocking the ACE2 receptor with antibodies or treating with cerebrospinal fluid derived from COVID-19 patients.

Immunostaining studies have confirmed that SARS-CoV-2 can infect organoids in various brain regions, including the hippocampus, hypothalamus, cortex, and midbrain [186]. The virus exhibits selective tropism to different cell types within these regions: astrocytes and neurons are generally susceptible, while choroid plexus epithelial cells show a significantly higher infection rate. To further investigate this phenomenon, researchers optimised the induction protocol and utilised iPSCs to construct more purified choroid plexus organoids. Their findings revealed that SARS-CoV-2 infection leads to an exacerbated inflammatory response and dysfunction in brain cells. Additional research demonstrated that the virus disrupts tight junctions between choroid plexus epithelial cells, resulting in cerebrospinal fluid leakage [187]. Moreover, cortical organoid models have shown that SARS-CoV-2 has a significant tropism for glial cells, particularly astrocytes, and triggers an immune response by activating the type I interferon pathway following infection [188]. Additionally, the genetic risk factor ApoE4 has been shown to enhance the susceptibility of PSC-derived neurons and astrocytes to infection, indicating its potential link to severe COVID-19 [189]. Mechanistic investigations have demonstrated that blocking the ACE2 receptor or inhibiting the DICER1 isoform of the double-stranded RNA cleaving enzyme can substantially decrease infection rates in organoids [185, 190].

Traditional brain organoid models lack a vascular system and a fully developed BBB, which may not accurately replicate the in vivo viral infection pathways or load dynamics [186]. Therefore, it is crucial to discern whether SARS-CoV-2-induced neurological damage arises from direct infection of brain cells or represents a secondary consequence of systemic inflammatory responses. Wang et al. developed a microphysiological system that integrates alveolar and BBB chips [117]. This system revealed minimal effects on the BBB component when directly exposed to SARS-CoV-2 viral particles. In contrast, the medium conditioned by infected alveolar chips significantly impacted the BBB chip, resulting in endothelial dysfunction, pericyte detachment, and neuroinflammation.

In summary, organoid models provide a robust platform for elucidating the mechanisms of viral invasion and pathogenesis within the nervous system. The rapidly evolving BBB chips and microphysiological systems overcome the limitations of conventional organoids and uncover inter-organ interactions during viral infections in the human body.

### Intestinal organoids

COVID-19 patients frequently exhibit gastrointestinal symptoms, including vomiting, diarrhea, and abdominal pain [191]. Research using PSC-derived intestinal organoids, encompassing the small intestine, colon, and ileum, has demonstrated that these cells are susceptible to SARS-CoV-2 and can support viral replication, indicating that the gastrointestinal tract is a significant target organ for the virus [136, 192]. Experimental evidence shows that SARS-CoV-2 infection induces intestinal organoid degeneration, while antiviral drugs such as Raltegravir and FDA-approved inhibitors effectively suppress viral replication [136, 193]. In addition, the blocking effect of antibodies or peptides targeting IFITM proteins on viral invasion was demonstrated in intestinal organoids [194]. The gene-edited intestinal organoid platform verified that TMPRSS2 and ACE2 regulate viral entry into intestinal organoids [140]. Moreover, the virus weakens the innate immune response by inhibiting interferon autocrine signaling, thereby preventing neighboring cells from activating interferon-stimulated genes [195, 196].

### Liver organoids

COVID-19 patients frequently exhibit abnormal liver function, characterized by elevated total bilirubin levels and altered alanine aminotransferase activity [197]. To explore the impact of SARS-CoV-2 on liver function, Zhao et al. employed an organoid model derived from hepatobiliary progenitor cells. They discovered that ACE2 +/TMPRSS2 + cholangiocytes were highly susceptible to SARS-CoV-2 infection, leading to direct cholangiocyte damage and subsequent bile acid accumulation [118]. These findings may elucidate the underlying mechanism of the liver dysfunction observed clinically. Notably, hepatobiliary organoids obtained from patients with nonalcoholic steatohepatitis demonstrated increased resistance to pseudovirus infection [198].

### Kidney organoids

Some SARS-CoV-2 infection cases may be complicated by acute kidney injury, kidney failure or fibrosis [130], prompting researchers to investigate the viral impact on renal tissue using kidney organoids derived from PSCs. Xia et al. demonstrated that renal proximal tubular epithelial cells in 3D kidney organoids express ACE2 at levels approximately twice as high as those observed in 2D cultures [199], underscoring the superior ability of 3D models to replicate COVID-19 pathology. Given that human ACE2 effectively suppressed SARS-CoV-2 replication in a dose-dependent manner, Monteil et al. attempted to inhibit SARS-CoV-2 infection in kidney organoids using human recombinant soluble ACE2 and achieved success [200].

### Heart organoids

The heart is a frequently affected organ in SARS-CoV-2 infection [201], with multiple cell types displaying pro-inflammatory transcriptional alterations in infected individuals [202]. However, research employing heart organoids for modelling infection remains limited. Thomas, D. et al. developed heart organoids from iPSC-derived endothelial cells and cardiomyocytes [203], which exhibit significant dysfunction following SARS-CoV-2 exposure, primarily driven by CCL2 secretion by endothelial cells. Further analysis demonstrated that CCL2-induced oxidative stress can result in post-translational modifications of cardiac proteins, thereby contributing to cardiac dysfunction. These findings underscore the crucial importance of early monitoring of vascular health in individuals with long COVID.

### Organ-on-chip and microfluidics system

Since the human body is a dynamically equilibrated system incorporating a variety of organic and physiological microenvironments, robust virus infection often involves interconnected changes in multiple systems and organoids. Organ-on-a-chip technology, which comprises microfluidic devices for culturing cells and tissues in continuously perfused chambers, offers dynamic and controlled microenvironments, enables integration of immune cells and multicellular interactions, and replicates the host response to virus infection. This technology is well-suited for studying the underlying mechanisms of pathology and virus-host interactions, drug resistance, and development of new antiviral therapies [204].

Human lung microarray has been employed to model virus infections and evaluate potential therapeutic agents. In an alveolar cell microarray with SARS-CoV-2 infection, tocilizumab was shown to mitigate barrier damage, which was accomplished by suppressing the inflammatory response rather than controlling viral replication [205]. In bronchial microarray with SARS-CoV-2 pseudotyped virus infection, amodiaquine and toremifene were identified as potential viral entry inhibitors [206]. Similar therapeutic effects of amodiaquine were also observed in the hamster model, suggesting that organ-on-a-chip systems may offer a viable alternative to animal models for discovering and evaluating drugs against infectious diseases. Interestingly, only two out of seven approved inhibitors of SARS-CoV-2 entry, amodiaquine and toremifene, were demonstrated to be effective in lung-on-a-chip. However, all these inhibitors were pre-screened using conventional 2D culture methods of the Huh-7 cell line, which is commonly used for in vitro screening of antiviral agents.

## Conclusion and outlook

The advancement of organoid technology has significantly propelled progress in virology research and expedited the process of clinical translation. Previous research on respiratory viruses and the development of treatment strategies has revealed several limitations in addressing the challenges posed by the SARS-CoV-2 pandemic. In the context of pathogen investigations, traditional animal models frequently exhibit insufficiencies in rapidly elucidating the intricate interactions between viruses and their host organisms. When it comes to the development of treatments, current platforms for vaccines and antiviral therapies are not always capable of providing timely protection, particularly for vulnerable populations, such as individuals with compromised immune systems, who are at a higher risk of hospitalization [155, 207, 208]. Most importantly, existing preclinical evaluation methods struggle to meet the urgent demand for validating interventions against potential future threats of "Disease X". Addressing these challenges calls for a reconsideration of the experimental phase by employing more human-relevant disease models that can effectively integrate the simulation of disease responses, the discovery of novel therapies, and the facilitation of personalized protective strategies. These unmet needs represent a distinctive opportunity to advance organoid technology into a robust and transformative platform for future biomedical research.

Currently, the development of organoids is becoming increasingly sophisticated, with continuous advancements in matrix materials and construction techniques. The microenvironment of the neural-immune-vascular system is being reconstructed with greater biological relevance, and the integration of multiple organoids reflects improved inter-organ communication and stress coordination, all of which aim to enhance simulation fidelity. Nevertheless, several challenges remain. First, it remains difficult to accurately replicate the anatomical continuity of complex organs using organoid models. For example, due to the distinct infection characteristics between the airway and alveolar epithelium in SARS-CoV-2 [209], most research is limited to constructing airway organoids and alveolar organoids separately. Furthermore, the investigation of multi-organ pathologies induced by SARS-CoV-2 is limited, particularly concerning the potential interactions and communication among affected organs, which remain poorly understood. Second, there is currently no effective approach to simulate the excessive inflammatory response triggered by viral infections and its interaction with the host immune system. Encouragingly, increased investment and interdisciplinary collaboration is driving progress in overcoming these technical barriers. For instance, by further exploring the differentiation patterns of stem cells, Koike H et al. report the formation of distinct yet interconnected organ structures resembling the liver, bile ducts, and pancreas, with continuous patterning and dynamic morphogenesis from a three-dimensional culture of human pluripotent stem cells [210], providing inspiration for the construction of multi-organ integration models of endodermal origin. By utilizing microfluidic systems, chips containing different organoids can be connected in series to enable the integration and precise regulation of microenvironments [211]. The resulting multi-organ model, also referred to as a micro-physiological system [212], has been employed in drug discovery and development. This system allows for systematic investigations of the effects of drugs and other stimuli across the entire multi-organ network, making it a promising tool for studying multi-organ infectious viruses such as SARS-CoV-2.

However, organoids still face significant challenges before they can be broadly adopted in clinical practice. It is critical to establish evaluation criteria and reach an industry-wide consensus on organoid construction protocols, as well as on the validation of morphological and functional properties, and on the reproducibility and consistency of experimental outcomes. These elements form the foundational framework for the clinical application of organoids. To facilitate clinical translation, a four-dimensional standard system might be developed: consensus category (identifying applicable scenarios and establishing normative frameworks), quality control category (standardising morphological and functional validation), technology category (optimising culture and detection methodologies), and application category (developing guidelines for pharmacological and vaccine evaluation). Looking ahead, when organoids are deployed in clinical settings, further improvements in simulation fidelity and the promotion of large-scale production to reduce costs will be essential. Only by balancing model accuracy with clinical feasibility can organoids become a core platform for next-generation antiviral drug development and personalised treatment planning, offering transformative tools for the prevention and control of respiratory infectious diseases.

## Data Availability

No new data were generated or analyzed in support of this review.

## Abbreviations

SARS-CoV-2: Severe acute respiratory syndrome coronavirus 2
RSV: Respiratory syncytial virus
ARDS: Acute respiratory distress syndrome
ECM: Extracellular matrix
ESCs: Embryonic stem cells
PSCs: Pluripotent stem cells
ASCs: Adult stem cells
iPSCs: Induced pluripotent stem cells
AT2: Alveolar type 2 cells
ALI: Air–liquid interface
BBB: Blood–brain barrier
AI: Artificial intelligence
AT1: Type I alveolar cells
ALOs: Airway-lung organoids
STR: Short tandem repeat
SNP: Nucleotide polymorphism
H&E: Hematoxylin and Eosin
scRNA-seq: Single-cell RNA sequencing
MERS-Co: Middle East respiratory syndrome coronavirus
OPAB: Organotypic cultures of human cortical brain explants
EKG: Electrocardiogram
PNEC: Pulmonary neuroendocrine cells

## References

[1] Baker RE, Mahmud AS, Miller IF, Rajeev M, Rasambainarivo F, Rice BL, et al. Infectious disease in an era of global change. Nat Rev Microbiol. 2022;20(4):193–205. 10.1038/s41579-021-00639-z. 34646006 10.1038/s41579-021-00639-z PMC8513385

[2] Bloom JD, Beichman AC, Neher RA, Harris K. Evolution of the SARS-CoV-2 Mutational Spectrum. Mol Biol Evol. 2023;40(4). 10.1093/molbev/msad085. 10.1093/molbev/msad085 PMC10124870 37039557

[3] Honce R, Schultz-Cherry S. Looking beyond the H5 avian influenza viruses. Cell. 2023;186(19):4003–4. 10.1016/j.cell.2023.08.014. 37714131 10.1016/j.cell.2023.08.014

[4] Krammer F. The human antibody response to influenza a virus infection and vaccination. Nat Rev Immunol. 2019;19(6):383–97. 10.1038/s41577-019-0143-6. 30837674 10.1038/s41577-019-0143-6

[5] Bos L, Ware LB. Acute respiratory distress syndrome: causes, pathophysiology, and phenotypes. Lancet. 2022;400(10358):1145–56. 10.1016/S0140-6736(22)01485-4. 36070787 10.1016/S0140-6736(22)01485-4

[6] Gorman EA, O’Kane CM, Mcauley DF. Acute respiratory distress syndrome in adults: diagnosis, outcomes, long-term sequelae, and management. Lancet. 2022;400(10358):1157–70. 10.1016/S0140-6736(22)01439-8. 36070788 10.1016/S0140-6736(22)01439-8

[7] Liu YN, Zhang YF, Xu Q, Qiu Y, Lu QB, Wang T, et al. Infection and co-infection patterns of community-acquired pneumonia in patients of different ages in China from 2009 to 2020: a national surveillance study. Lancet Microbe. 2023;4(5):e330–9. 10.1016/S2666-5247(23)00031-9. 37001538 10.1016/S2666-5247(23)00031-9 PMC12514336

[8] Nair GB, Niederman MS. Updates on community acquired pneumonia management in the ICU. Pharmacol Ther. 2021;217:107663. 10.1016/j.pharmthera.2020.107663. 32805298 10.1016/j.pharmthera.2020.107663 PMC7428725

[9] Chen Z, Yuan Y, Hu Q, Zhu A, Chen F, Li S, et al. SARS-CoV-2 immunity in animal models. Cell Mol Immunol. 2024;21(2):119–33. 10.1038/s41423-023-01122-w. 38238440 10.1038/s41423-023-01122-w PMC10806257

[10] Munoz-Fontela C, Dowling WE, Funnell S, Gsell PS, Riveros-Balta AX, Albrecht RA, et al. Animal models for COVID-19. Nature. 2020;586(7830):509–15. 10.1038/s41586-020-2787-6. 32967005 10.1038/s41586-020-2787-6 PMC8136862

[11] Kiesslich S, Kamen AA. Vero cell upstream bioprocess development for the production of viral vectors and vaccines. Biotechnol Adv. 2020;44:107608. 10.1016/j.biotechadv.2020.107608. 32768520 10.1016/j.biotechadv.2020.107608 PMC7405825

[12] Duval K, Grover H, Han LH, Mou Y, Pegoraro AF, Fredberg J, et al. Modeling Physiological Events in 2D vs. 3D Cell Culture. Physiology (Bethesda). 2017;32(4):266–77. 10.1152/physiol.00036.2016. 28615311 10.1152/physiol.00036.2016 PMC5545611

[13] Lancaster MA, Knoblich JA. Organogenesis in a dish: modeling development and disease using organoid technologies. Science. 2014;345(6194):1247125. 10.1126/science.1247125. 25035496 10.1126/science.1247125

[14] Bose S, Clevers H, Shen X. Promises and Challenges of Organoid-Guided Precision Medicine. Med (N Y). 2021;2(9):1011–26. 10.1016/j.medj.2021.08.005. 10.1016/j.medj.2021.08.005 PMC8492003 34617071

[15] Bock C, Boutros M, Camp JG, Clarke L, Clevers H, Knoblich JA, et al. The Organoid Cell Atlas. Nat Biotechnol. 2021;39(1):13–7. 10.1038/s41587-020-00762-x. 33384458 10.1038/s41587-020-00762-x PMC7801253

[16] Sato T, Vries RG, Snippert HJ, van de Wetering M, Barker N, Stange DE, et al. Single Lgr5 stem cells build crypt-villus structures in vitro without a mesenchymal niche. Nature. 2009;459(7244):262–5. 10.1038/nature07935. 19329995 10.1038/nature07935

[17] Blutt SE, Estes MK. Organoid Models for Infectious Disease. Annu Rev Med. 2022;73:167–82. 10.1146/annurev-med-042320-023055. 34644153 10.1146/annurev-med-042320-023055 PMC8887824

[18] Wilson HV. A new method by which sponges may be artificially reared. Science. 1907;25(649):912–5. 10.1126/science.25.649.912. 17842577 10.1126/science.25.649.912

[19] Steinberg MS. Reconstruction of Tissues by Dissociated Cells. Science. 1963;141(3579):401–8. 10.1126/science.141.3579.401. 13983728 10.1126/science.141.3579.401

[20] Moscona AA. Tissues from dissociated cells. Sci Am. 1959;200(5):132–48. 13646649 10.1038/scientificamerican0559-132

[21] Rheinwald JG, Green H. Serial cultivation of strains of human epidermal keratinocytes: the formation of keratinizing colonies from single cells. Cell. 1975;6(3):331–43. 10.1016/s0092-8674(75)80001-8. 1052771 10.1016/s0092-8674(75)80001-8

[22] Emerman JT, Pitelka DR. Maintenance and induction of morphological differentiation in dissociated mammary epithelium on floating collagen membranes. In Vitro. 1977;13(5):316–28. 10.1007/BF02616178. 559643 10.1007/BF02616178

[23] Thomson JA, Itskovitz-Eldor J, Shapiro SS, Waknitz MA, Swiergiel JJ, Marshall VS, et al. Embryonic stem cell lines derived from human blastocysts. Science. 1998;282(5391):1145–7. 10.1126/science.282.5391.1145. 9804556 10.1126/science.282.5391.1145

[24] Yu J, Vodyanik MA, Smuga-Otto K, Antosiewicz-Bourget J, Frane JL, Tian S, et al. Induced pluripotent stem cell lines derived from human somatic cells. Science. 2007;318(5858):1917–20. 10.1126/science.1151526. 18029452 10.1126/science.1151526

[25] Aisenbrey EA, Murphy WL. Synthetic alternatives to Matrigel. Nat Rev Mater. 2020;5(7):539–51. 10.1038/s41578-020-0199-8. 32953138 10.1038/s41578-020-0199-8 PMC7500703

[26] Avery K, Chen X. Integration of bioprinting advances and biomechanical strategies for*in vitro* lung modelling. Biofabrication. 2024;17(1). 10.1088/1758-5090/ad91e2. 10.1088/1758-5090/ad91e2 39536463

[27] Humphreys BD. Bioprinting better kidney organoids. Nat Mater. 2021;20(2):128–30. 10.1038/s41563-020-00881-5. 33504983 10.1038/s41563-020-00881-5

[28] She W, Shen C, Xue Z, Zhang B, Zhang G, Meng Q. Hydrogel Strain Sensors for Integrating Into Dynamic Organ-on-a-Chip. Small. 2025;21(7):e2407704. 10.1002/smll.202407704. 39846814 10.1002/smll.202407704

[29] Takebe T, Zhang B, Radisic M. Synergistic Engineering: Organoids Meet Organs-on-a-Chip. Cell Stem Cell. 2017;21(3):297–300. 10.1016/j.stem.2017.08.016. 28886364 10.1016/j.stem.2017.08.016

[30] Artegiani B, Hendriks D. Organoids from pluripotent stem cells and human tissues: When two cultures meet each other. Dev Cell. 2025;60(4):493–511. 10.1016/j.devcel.2025.01.005. 39999776 10.1016/j.devcel.2025.01.005

[31] Han Y, Yang L, Lacko LA, Chen S. Human organoid models to study SARS-CoV-2 infection. Nat Methods. 2022;19(4):418–28. 10.1038/s41592-022-01453-y. 35396481 10.1038/s41592-022-01453-y

[32] Huang SX, Green MD, de Carvalho AT, Mumau M, Chen YW, D’Souza SL, et al. The in vitro generation of lung and airway progenitor cells from human pluripotent stem cells. Nat Protoc. 2015;10(3):413–25. 10.1038/nprot.2015.023. 25654758 10.1038/nprot.2015.023 PMC4654940

[33] Bock C, Kiskinis E, Verstappen G, Gu H, Boulting G, Smith ZD, et al. Reference Maps of human ES and iPS cell variation enable high-throughput characterization of pluripotent cell lines. Cell. 2011;144(3):439–52. 10.1016/j.cell.2010.12.032. 21295703 10.1016/j.cell.2010.12.032 PMC3063454

[34] Yamanaka S. Induced pluripotent stem cells: past, present, and future. Cell Stem Cell. 2012;10(6):678–84. 10.1016/j.stem.2012.05.005. 22704507 10.1016/j.stem.2012.05.005

[35] Salgueiro L, Kummer S, Sonntag-Buck V, Weiss A, Schneider MA, Krausslich HG, et al. Generation of Human Lung Organoid Cultures from Healthy and Tumor Tissue to Study Infectious Diseases. J Virol. 2022;96(7):e0009822. 10.1128/jvi.00098-22. 35285684 10.1128/jvi.00098-22 PMC9006928

[36] Yamamoto Y, Gotoh S, Korogi Y, Seki M, Konishi S, Ikeo S, et al. Long-term expansion of alveolar stem cells derived from human iPS cells in organoids. Nat Methods. 2017;14(11):1097–106. 10.1038/nmeth.4448. 28967890 10.1038/nmeth.4448

[37] Prentice DA. Adult Stem Cells. Circ Res. 2019;124(6):837–9. 10.1161/CIRCRESAHA.118.313664. 30870122 10.1161/CIRCRESAHA.118.313664

[38] Barkauskas CE, Cronce MJ, Rackley CR, Bowie EJ, Keene DR, Stripp BR, et al. Type 2 alveolar cells are stem cells in adult lung. J Clin Invest. 2013;123(7):3025–36. 10.1172/JCI68782. 23921127 10.1172/JCI68782 PMC3696553

[39] van der Vaart J, Clevers H. Airway organoids as models of human disease. J Intern Med. 2021;289(5):604–13. 10.1111/joim.13075. 32350962 10.1111/joim.13075

[40] Vazquez-Armendariz AI, Tata PR. Recent advances in lung organoid development and applications in disease modeling. J Clin Invest. 2023;133(22). 10.1172/JCI170500. 10.1172/JCI170500 PMC10645385 37966116

[41] Katsura H, Sontake V, Tata A, Kobayashi Y, Edwards CE, Heaton BE, et al. Human Lung Stem Cell-Based Alveolospheres Provide Insights into SARS-CoV-2-Mediated Interferon Responses and Pneumocyte Dysfunction. Cell Stem Cell. 2020;27(6):890–904. 10.1016/j.stem.2020.10.005. 33128895 10.1016/j.stem.2020.10.005 PMC7577733

[42] Salahudeen AA, Choi SS, Rustagi A, Zhu J, van Unen V, de la O SM, et al. Progenitor identification and SARS-CoV-2 infection in human distal lung organoids. Nature. 2020;588(7839):670–5. 10.1038/s41586-020-3014-1. 33238290 10.1038/s41586-020-3014-1 PMC8003326

[43] Du L, Bouzidi MS, Gala A, Deiter F, Billaud JN, Yeung ST, et al. Human galectin-9 potently enhances SARS-CoV-2 replication and inflammation in airway epithelial cells. J Mol Cell Biol. 2023;15(4). 10.1093/jmcb/mjad030. 10.1093/jmcb/mjad030 PMC10668544 37127426

[44] Wang Y, Thaler M, Salgado-Benvindo C, Ly N, Leijs AA, Ninaber DK, et al. SARS-CoV-2-infected human airway epithelial cell cultures uniquely lack interferon and immediate early gene responses caused by other coronaviruses. Clin Transl Immunology. 2024;13(4):e1503. 10.1002/cti2.1503. 38623540 10.1002/cti2.1503 PMC11017760

[45] Silva S, Bicker J, Falcao A, Fortuna A. Air-liquid interface (ALI) impact on different respiratory cell cultures. Eur J Pharm Biopharm. 2023;184:62–82. 10.1016/j.ejpb.2023.01.013. 36696943 10.1016/j.ejpb.2023.01.013

[46] Chiu MC, Li C, Yu Y, Liu X, Huang J, Wan Z, et al. Establishing Bipotential Human Lung Organoid Culture System and Differentiation to Generate Mature Alveolar and Airway Organoids. Bio Protoc. 2023;13(8):e4657. 10.21769/BioProtoc.4657. 10.21769/BioProtoc.4657 PMC10127040 37113328

[47] Li C, Chiu MC, Yu Y, Liu X, Xiao D, Huang J, et al. Establishing Human Lung Organoids and Proximal Differentiation to Generate Mature Airway Organoids. J Vis Exp. 2022;(181). 10.3791/63684. 10.3791/63684 35404361

[48] Castaneda DC, Jangra S, Yurieva M, Martinek J, Callender M, Coxe M, et al. Protocol for establishing primary human lung organoid-derived air-liquid interface cultures from cryopreserved human lung tissue. STAR Protoc. 2023;4(4):102735. 10.1016/j.xpro.2023.102735. 37991921 10.1016/j.xpro.2023.102735 PMC10696416

[49] Lamers MM, van der Vaart J, Knoops K, Riesebosch S, Breugem TI, Mykytyn AZ, et al. An organoid-derived bronchioalveolar model for SARS-CoV-2 infection of human alveolar type II-like cells. EMBO J. 2021;40(5):e105912. 10.15252/embj.2020105912. 10.15252/embj.2020105912 PMC7883112 33283287

[50] Kim J, Eo EY, Kim B, Lee H, Kim J, Koo BK, et al. Transcriptomic Analysis of Air-Liquid Interface Culture in Human Lung Organoids Reveals Regulators of Epithelial Differentiation. Cells. 2024;13(23). 10.3390/cells13231991. 10.3390/cells13231991 PMC11639892 39682739

[51] Broutier L, Andersson-Rolf A, Hindley CJ, Boj SF, Clevers H, Koo BK, et al. Culture and establishment of self-renewing human and mouse adult liver and pancreas 3D organoids and their genetic manipulation. Nat Protoc. 2016;11(9):1724–43. 10.1038/nprot.2016.097. 27560176 10.1038/nprot.2016.097

[52] Chen YW, Huang SX, de Carvalho A, Ho SH, Islam MN, Volpi S, et al. A three-dimensional model of human lung development and disease from pluripotent stem cells. Nat Cell Biol. 2017;19(5):542–9. 10.1038/ncb3510. 28436965 10.1038/ncb3510 PMC5777163

[53] Sato T, Stange DE, Ferrante M, Vries RG, Van Es JH, Van den Brink S, et al. Long-term expansion of epithelial organoids from human colon, adenoma, adenocarcinoma, and Barrett’s epithelium. Gastroenterology. 2011;141(5):1762–72. 10.1053/j.gastro.2011.07.050. 21889923 10.1053/j.gastro.2011.07.050

[54] Wang F, Scoville D, He XC, Mahe MM, Box A, Perry JM, et al. Isolation and characterization of intestinal stem cells based on surface marker combinations and colony-formation assay. Gastroenterology. 2013;145(2):383–95. 10.1053/j.gastro.2013.04.050. 23644405 10.1053/j.gastro.2013.04.050 PMC3781924

[55] Witek MA, Freed IM, Soper SA. Cell Separations and Sorting. Anal Chem. 2020;92(1):105–31. 10.1021/acs.analchem.9b05357. 31808677 10.1021/acs.analchem.9b05357 PMC7469080

[56] Maharjan S, Ma C, Singh B, Kang H, Orive G, Yao J, et al. Advanced 3D imaging and organoid bioprinting for biomedical research and therapeutic applications. Adv Drug Deliv Rev. 2024;208:115237. 10.1016/j.addr.2024.115237. 38447931 10.1016/j.addr.2024.115237 PMC11031334

[57] Kozlowski MT, Crook CJ, Ku HT. Towards organoid culture without Matrigel. Commun Biol. 2021;4(1):1387. 10.1038/s42003-021-02910-8. 34893703 10.1038/s42003-021-02910-8 PMC8664924

[58] Kratochvil MJ, Seymour AJ, Li TL, Pasca SP, Kuo CJ, Heilshorn SC. Engineered materials for organoid systems. Nat Rev Mater. 2019;4(9):606–22. 10.1038/s41578-019-0129-9. 33552558 10.1038/s41578-019-0129-9 PMC7864216

[59] Urciuolo A, Giobbe GG, Dong Y, Michielin F, Brandolino L, Magnussen M, et al. Hydrogel-in-hydrogel live bioprinting for guidance and control of organoids and organotypic cultures. Nat Commun. 2023;14(1):3128. 10.1038/s41467-023-37953-4. 37253730 10.1038/s41467-023-37953-4 PMC10229611

[60] Orkin RW, Gehron P, Mcgoodwin EB, Martin GR, Valentine T, Swarm R. A murine tumor producing a matrix of basement membrane. J Exp Med. 1977;145(1):204–20. 10.1084/jem.145.1.204. 830788 10.1084/jem.145.1.204 PMC2180589

[61] Kleinman HK, Martin GR. Matrigel: basement membrane matrix with biological activity. Semin Cancer Biol. 2005;15(5):378–86. 10.1016/j.semcancer.2005.05.004. 15975825 10.1016/j.semcancer.2005.05.004

[62] Zhao KY, Du YX, Cao HM, Su LY, Su XL, Li X. The biological macromolecules constructed Matrigel for cultured organoids in biomedical and tissue engineering. Colloids Surf B Biointerfaces. 2025;247:114435. 10.1016/j.colsurfb.2024.114435. 39647422 10.1016/j.colsurfb.2024.114435

[63] Patel M, Lee HJ, Park S, Kim Y, Jeong B. Injectable thermogel for 3D culture of stem cells. Biomaterials. 2018;159:91–107. 10.1016/j.biomaterials.2018.01.001. 29316455 10.1016/j.biomaterials.2018.01.001

[64] Kaur S, Kaur I, Rawal P, Tripathi DM, Vasudevan A. Non-matrigel scaffolds for organoid cultures. Cancer Lett. 2021;504:58–66. 10.1016/j.canlet.2021.01.025. 33582211 10.1016/j.canlet.2021.01.025

[65] Cao H, Duan L, Zhang Y, Cao J, Zhang K. Current hydrogel advances in physicochemical and biological response-driven biomedical application diversity. Signal Transduct Target Ther. 2021;6(1):426. 10.1038/s41392-021-00830-x. 34916490 10.1038/s41392-021-00830-x PMC8674418

[66] Gan Z, Qin X, Liu H, Liu J, Qin J. Recent advances in defined hydrogels in organoid research. Bioact Mater. 2023;28:386–401. 10.1016/j.bioactmat.2023.06.004. 37334069 10.1016/j.bioactmat.2023.06.004 PMC10273284

[67] Lou J, Mooney DJ. Chemical strategies to engineer hydrogels for cell culture. Nat Rev Chem. 2022;6(10):726–44. 10.1038/s41570-022-00420-7. 37117490 10.1038/s41570-022-00420-7

[68] Li C, An N, Song Q, Hu Y, Yin W, Wang Q, et al. Enhancing organoid culture: harnessing the potential of decellularized extracellular matrix hydrogels for mimicking microenvironments. J Biomed Sci. 2024;31(1):96. 10.1186/s12929-024-01086-7. 39334251 10.1186/s12929-024-01086-7 PMC11429032

[69] Li G, Liu S, Chen W, Jiang Z, Luo Y, Wang D, et al. Acellularized Uvea Hydrogel as Novel Injectable Platform for Cell-Based Delivering Treatment of Retinal Degeneration and Optimizing Retinal Organoids Inducible System. Adv Healthc Mater. 2022;11(23):e2202114. 10.1002/adhm.202202114. 36189847 10.1002/adhm.202202114

[70] Vyas D, Baptista PM, Brovold M, Moran E, Gaston B, Booth C, et al. Self-assembled liver organoids recapitulate hepatobiliary organogenesis in vitro. Hepatology. 2018;67(2):750–61. 10.1002/hep.29483. 28834615 10.1002/hep.29483 PMC5825235

[71] Quan Y, Li J, Cai J, Liao Y, Zhang Y, Lu F. Transplantation of beige adipose organoids fabricated using adipose acellular matrix hydrogel improves metabolic dysfunction in high-fat diet-induced obesity and type 2 diabetes mice. J Cell Physiol. 2024;239(4):e31191. 10.1002/jcp.31191. 38219044 10.1002/jcp.31191

[72] Deniz DI, Yeo M, Castaneda DC, Callender M, Horvath M, Mo Z, et al. High-throughput bioprinting of the nasal epithelium using patient-derived nasal epithelial cells. Biofabrication. 2023;15(4). 10.1088/1758-5090/aced23. 10.1088/1758-5090/aced23 PMC10424246 37536321

[73] Chen AJ, Dong J, Yuan XH, Bo H, Li SZ, Wang C, et al. Anti-H7N9 avian influenza a virus activity of interferon in pseudostratified human airway epithelium cell cultures. Virol J. 2019;16(1):44. 10.1186/s12985-019-1146-4. 30944006 10.1186/s12985-019-1146-4 PMC6448296

[74] Hynes RO. The extracellular matrix: not just pretty fibrils. Science. 2009;326(5957):1216–9. 10.1126/science.1176009. 19965464 10.1126/science.1176009 PMC3536535

[75] Kishimoto K, Iwasawa K, Sorel A, Ferran-Heredia C, Han L, Morimoto M, et al. Directed differentiation of human pluripotent stem cells into diverse organ-specific mesenchyme of the digestive and respiratory systems. Nat Protoc. 2022;17(11):2699–719. 10.1038/s41596-022-00733-3. 35978039 10.1038/s41596-022-00733-3 PMC9633385

[76] Luo Z, Zhang S, Pan J, Shi R, Liu H, Lyu Y, et al. Time-responsive osteogenic niche of stem cells: a sequentially triggered, dual-peptide loaded, alginate hybrid system for promoting cell activity and osteo-differentiation. Biomaterials. 2018;163:25–42. 10.1016/j.biomaterials.2018.02.025. 29452946 10.1016/j.biomaterials.2018.02.025

[77] Rocha FG, Sundback CA, Krebs NJ, Leach JK, Mooney DJ, Ashley SW, et al. The effect of sustained delivery of vascular endothelial growth factor on angiogenesis in tissue-engineered intestine. Biomaterials. 2008;29(19):2884–90. 10.1016/j.biomaterials.2008.03.026. 18396329 10.1016/j.biomaterials.2008.03.026 PMC2685178

[78] Allazetta S, Lutolf MP. Stem cell niche engineering through droplet microfluidics. Curr Opin Biotechnol. 2015;35:86–93. 10.1016/j.copbio.2015.05.003. 26051090 10.1016/j.copbio.2015.05.003

[79] Hofer M, Lutolf MP. Engineering organoids. Nat Rev Mater. 2021;6(5):402–20. 10.1038/s41578-021-00279-y. 33623712 10.1038/s41578-021-00279-y PMC7893133

[80] Eiraku M, Takata N, Ishibashi H, Kawada M, Sakakura E, Okuda S, et al. Self-organizing optic-cup morphogenesis in three-dimensional culture. Nature. 2011;472(7341):51–6. 10.1038/nature09941. 21475194 10.1038/nature09941

[81] Yi SA, Zhang Y, Rathnam C, Pongkulapa T, Lee KB. Bioengineering Approaches for the Advanced Organoid Research. Adv Mater. 2021;33(45):e2007949. 10.1002/adma.202007949. 34561899 10.1002/adma.202007949 PMC8682947

[82] Zan GX, Qin YC, Xie WW, Gao CQ, Yan HC, Wang XQ, et al. Heat stress disrupts intestinal stem cell migration and differentiation along the crypt-villus axis through FAK signaling. Biochim Biophys Acta Mol Cell Res. 2023;1870(3):119431. 10.1016/j.bbamcr.2023.119431. 36632926 10.1016/j.bbamcr.2023.119431

[83] Li P, Wang Y, Lamers MM, Lavrijsen M, Iriondo C, de Vries AC, et al. Recapitulating infection, thermal sensitivity and antiviral treatment of seasonal coronaviruses in human airway organoids. EBioMedicine. 2022;81:104132. 10.1016/j.ebiom.2022.104132. 35779493 10.1016/j.ebiom.2022.104132 PMC9240613

[84] Chugh M, Munjal A, Megason SG. Hydrostatic pressure as a driver of cell and tissue morphogenesis. Semin Cell Dev Biol. 2022;131:134–45. 10.1016/j.semcdb.2022.04.021. 35534334 10.1016/j.semcdb.2022.04.021 PMC9529827

[85] Kong D, Ryu JC, Shin N, Lee SE, Kim NG, Kim HY, et al. In Vitro Modeling of Atherosclerosis Using iPSC-Derived Blood Vessel Organoids. Adv Healthc Mater. 2025;14(1):e2400919. 10.1002/adhm.202400919. 39580678 10.1002/adhm.202400919

[86] Li SR, Gulieva RE, Helms L, Cruz NM, Vincent T, Fu H, et al. Glucose absorption drives cystogenesis in a human organoid-on-chip model of polycystic kidney disease. Nat Commun. 2022;13(1):7918. 10.1038/s41467-022-35537-2. 36564419 10.1038/s41467-022-35537-2 PMC9789147

[87] Ma HL, Urbaczek AC, Zeferino RDSF, Bernal C, Rodrigues PJ, Carrilho E. Replicating endothelial shear stress in organ-on-a-chip for predictive hypericin photodynamic efficiency. Int J Pharm. 2023;634:122629. 10.1016/j.ijpharm.2023.122629. 36682507 10.1016/j.ijpharm.2023.122629

[88] Gjorevski N, Nikolaev M, Brown TE, Mitrofanova O, Brandenberg N, Delrio FW, et al. Tissue geometry drives deterministic organoid patterning. Science. 2022;375(6576):eaaw9021. 10.1126/science.aaw9021. 10.1126/science.aaw9021 PMC9131435 34990240

[89] Mcmurtrey RJ. Analytic Models of Oxygen and Nutrient Diffusion, Metabolism Dynamics, and Architecture Optimization in Three-Dimensional Tissue Constructs with Applications and Insights in Cerebral Organoids. Tissue Eng Part C Methods. 2016;22(3):221–49. 10.1089/ten.TEC.2015.0375. 26650970 10.1089/ten.tec.2015.0375 PMC5029285

[90] Gao D, Ernst AU, Wang X, Wang L, Liu W, Ma M. Engineering a Hierarchical Biphasic Gel for Subcutaneous Vascularization. Adv Healthc Mater. 2022;11(19):e2200922. 10.1002/adhm.202200922. 35894816 10.1002/adhm.202200922

[91] Zhang S, Kan EL, Kamm RD. Integrating functional vasculature into organoid culture: a biomechanical perspective. APL Bioeng. 2022;6(3):30401. 10.1063/5.0097967. 10.1063/5.0097967 PMC9262412 35813884

[92] Velasco S, Kedaigle AJ, Simmons SK, Nash A, Rocha M, Quadrato G, et al. Individual brain organoids reproducibly form cell diversity of the human cerebral cortex. Nature. 2019;570(7762):523–7. 10.1038/s41586-019-1289-x. 31168097 10.1038/s41586-019-1289-x PMC6906116

[93] Homan KA, Gupta N, Kroll KT, Kolesky DB, Skylar-Scott M, Miyoshi T, et al. Flow-enhanced vascularization and maturation of kidney organoids in vitro. Nat Methods. 2019;16(3):255–62. 10.1038/s41592-019-0325-y. 30742039 10.1038/s41592-019-0325-y PMC6488032

[94] Roos F, van Tienderen GS, Wu H, Bordeu I, Vinke D, Albarinos LM, et al. Human branching cholangiocyte organoids recapitulate functional bile duct formation. Cell Stem Cell. 2022;29(5):776–94. 10.1016/j.stem.2022.04.011. 35523140 10.1016/j.stem.2022.04.011

[95] Nikolaev M, Mitrofanova O, Broguiere N, Geraldo S, Dutta D, Tabata Y, et al. Homeostatic mini-intestines through scaffold-guided organoid morphogenesis. Nature. 2020;585(7826):574–8. 10.1038/s41586-020-2724-8. 32939089 10.1038/s41586-020-2724-8

[96] Maurat E, Raasch K, Leipold AM, Henrot P, Zysman M, Prevel R, et al. A novel in vitro tubular model to recapitulate features of distal airways: the bronchioid. Eur Respir J. 2024. 10.1183/13993003.00562-2024. 39231631 10.1183/13993003.00562-2024 PMC11627163

[97] Sun J, Ahmed I, Brown J, Khosrotehrani K, Shafiee A. The empowering influence of air-liquid interface culture on skin organoid hair follicle development. Burns Trauma. 2025;13:tkae070. 10.1093/burnst/tkae070. 10.1093/burnst/tkae070 PMC11736897 39822647

[98] Lee RE, Reidel B, Nelson MR, Macdonald JK, Kesimer M, Randell SH. Air-Liquid interface cultures to model drug delivery through the mucociliary epithelial barrier. Adv Drug Deliv Rev. 2023;198:114866. 10.1016/j.addr.2023.114866. 37196698 10.1016/j.addr.2023.114866 PMC10336980

[99] Papp D, Korcsmaros T, Hautefort I. Revolutionizing immune research with organoid-based co-culture and chip systems. Clin Exp Immunol. 2024;218(1):40–54. 10.1093/cei/uxae004. 38280212 10.1093/cei/uxae004 PMC11404127

[100] Kang JS, Lee Y, Lee Y, Gil D, Kim MJ, Wood C, et al. Generation of induced alveolar assembloids with functional alveolar-like macrophages. Nat Commun. 2025;16(1):3346. 10.1038/s41467-025-58450-w. 40199883 10.1038/s41467-025-58450-w PMC11978882

[101] Seo H, Han H, Lee Y, Noh Y, Cho S, Kim J. Human pluripotent stem cell-derived alveolar organoid with macrophages. Int J Mol Sci. 2022;23(16). 10.3390/ijms23169211 PMC9409017 36012471

[102] Yang L, Nilsson-Payant BE, Han Y, Jaffre F, Zhu J, Wang P, et al. Cardiomyocytes recruit monocytes upon SARS-CoV-2 infection by secreting CCL2. Stem Cell Reports. 2021;16(9):2274–88. 10.1016/j.stemcr.2021.07.012. 34403650 10.1016/j.stemcr.2021.07.012 PMC8289700

[103] Yang L, Han Y, Jaffre F, Nilsson-Payant BE, Bram Y, Wang P, et al. An Immuno-Cardiac Model for Macrophage-Mediated Inflammation in COVID-19 Hearts. Circ Res. 2021;129(1):33–46. 10.1161/CIRCRESAHA.121.319060. 33853355 10.1161/CIRCRESAHA.121.319060 PMC8225586

[104] Wagar LE, Salahudeen A, Constantz CM, Wendel BS, Lyons MM, Mallajosyula V, et al. Modeling human adaptive immune responses with tonsil organoids. Nat Med. 2021;27(1):125–35. 10.1038/s41591-020-01145-0. 33432170 10.1038/s41591-020-01145-0 PMC7891554

[105] Jiang S, Zhao H, Zhang W, Wang J, Liu Y, Cao Y, et al. An Automated Organoid Platform with Inter-organoid Homogeneity and Inter-patient Heterogeneity. Cell Rep Med. 2020;1(9):100161. 10.1016/j.xcrm.2020.100161. 33377132 10.1016/j.xcrm.2020.100161 PMC7762778

[106] Bai H, Si L, Jiang A, Belgur C, Zhai Y, Plebani R, et al. Mechanical control of innate immune responses against viral infection revealed in a human lung alveolus chip. Nat Commun. 2022;13(1):1928. 10.1038/s41467-022-29562-4. 35396513 10.1038/s41467-022-29562-4 PMC8993817

[107] Alimonti JB, Ribecco-Lutkiewicz M, Sodja C, Jezierski A, Stanimirovic DB, Liu Q, et al. Zika virus crosses an in vitro human blood brain barrier model. Fluids Barriers CNS. 2018;15(1):15. 10.1186/s12987-018-0100-y. 29759080 10.1186/s12987-018-0100-y PMC5952854

[108] Villenave R, Wales SQ, Hamkins-Indik T, Papafragkou E, Weaver JC, Ferrante TC, et al. Human Gut-On-a-Chip Supports Polarized Infection of Coxsackie B1 Virus in Vitro. PLoS ONE. 2017;12(2):e0169412. 10.1371/journal.pone.0169412. 28146569 10.1371/journal.pone.0169412 PMC5287454

[109] Cao T, Shao C, Yu X, Xie R, Yang C, Sun Y, et al. Biomimetic Alveolus-on-a-Chip for SARS-CoV-2 Infection Recapitulation. Research (Wash D C). 2022;2022:9819154. 10.34133/2022/9819154. 10.34133/2022/9819154 PMC8841031 35224503

[110] Deng S, Li C, Cao J, Cui Z, Du J, Fu Z, et al. Organ-on-a-chip meets artificial intelligence in drug evaluation. Theranostics. 2023;13(13):4526–58. 10.7150/thno.87266. 37649608 10.7150/thno.87266 PMC10465229

[111] Lin Z, Zou Z, Pu Z, Wu M, Zhang Y. Application of microfluidic technologies on COVID-19 diagnosis and drug discovery. Acta Pharm Sin B. 2023;13(7):2877–96. 10.1016/j.apsb.2023.02.014. 36855672 10.1016/j.apsb.2023.02.014 PMC9951611

[112] Yu M, Selvaraj SK, Liang-Chu MM, Aghajani S, Busse M, Yuan J, et al. A resource for cell line authentication, annotation and quality control. Nature. 2015;520(7547):307–11. 10.1038/nature14397. 25877200 10.1038/nature14397

[113] Liu Y, Chudgar N, Mastrogiacomo B, He D, Lankadasari MB, Bapat S, et al. A germline SNP in BRMS1 predisposes patients with lung adenocarcinoma to metastasis and can be ameliorated by targeting c-fos. Sci Transl Med. 2022;14(665):eabo1050. 10.1126/scitranslmed.abo1050. 10.1126/scitranslmed.abo1050 PMC9926934 36197962

[114] Kadur LMP, Sontake V, Tata A, Kobayashi Y, Macadlo L, Okuda K, et al. Human distal lung maps and lineage hierarchies reveal a bipotent progenitor. Nature. 2022;604(7904):111–9. 10.1038/s41586-022-04541-3. 35355018 10.1038/s41586-022-04541-3 PMC9169066

[115] Gao X, Bali AS, Randell SH, Hogan BL. GRHL2 coordinates regeneration of a polarized mucociliary epithelium from basal stem cells. J Cell Biol. 2015;211(3):669–82. 10.1083/jcb.201506014. 26527742 10.1083/jcb.201506014 PMC4639861

[116] Lancaster MA, Renner M, Martin CA, Wenzel D, Bicknell LS, Hurles ME, et al. Cerebral organoids model human brain development and microcephaly. Nature. 2013;501(7467):373–9. 10.1038/nature12517. 10.1038/nature12517 PMC3817409 23995685

[117] Wang P, Jin L, Zhang M, Wu Y, Duan Z, Guo Y, et al. Blood-brain barrier injury and neuroinflammation induced by SARS-CoV-2 in a lung-brain microphysiological system. Nat Biomed Eng. 2024;8(8):1053–68. 10.1038/s41551-023-01054-w. 37349391 10.1038/s41551-023-01054-w

[118] Zhao B, Ni C, Gao R, Wang Y, Yang L, Wei J, et al. Recapitulation of SARS-CoV-2 infection and cholangiocyte damage with human liver ductal organoids. Protein Cell. 2020;11(10):771–5. 10.1007/s13238-020-00718-6. 32303993 10.1007/s13238-020-00718-6 PMC7164704

[119] Du X, Chen Z, Li Q, Yang S, Jiang L, Yang Y, et al. Organoids revealed: morphological analysis of the profound next generation in-vitro model with artificial intelligence. Biodes Manuf. 2023;6(3):319–39. 10.1007/s42242-022-00226-y. 36713614 10.1007/s42242-022-00226-y PMC9867835

[120] Enrico A, Voulgaris D, Ostmans R, Sundaravadivel N, Moutaux L, Cordier A, et al. 3D Microvascularized Tissue Models by Laser-Based Cavitation Molding of Collagen. Adv Mater. 2022;34(11):e2109823. 10.1002/adma.202109823. 35029309 10.1002/adma.202109823

[121] Co JY, Margalef-Catala M, Monack DM, Amieva MR. Controlling the polarity of human gastrointestinal organoids to investigate epithelial biology and infectious diseases. Nat Protoc. 2021;16(11):5171–92. 10.1038/s41596-021-00607-0. 34663962 10.1038/s41596-021-00607-0 PMC8841224

[122] Hoffmann PC, Giandomenico SL, Ganeva I, Wozny MR, Sutcliffe M, Lancaster MA, et al. Electron cryo-tomography reveals the subcellular architecture of growing axons in human brain organoids. Elife. 2021;10. 10.7554/eLife.70269. 10.7554/eLife.70269 PMC8547956 34698018

[123] Mosa MH, Nicolle O, Maschalidi S, Sepulveda FE, Bidaud-Meynard A, Menche C, et al. Dynamic Formation of Microvillus Inclusions During Enterocyte Differentiation in Munc18–2-Deficient Intestinal Organoids. Cell Mol Gastroenterol Hepatol. 2018;6(4):477–93. 10.1016/j.jcmgh.2018.08.001. 30364784 10.1016/j.jcmgh.2018.08.001 PMC6198061

[124] Perez-Gonzalez C, Ceada G, Greco F, Matejcic M, Gomez-Gonzalez M, Castro N, et al. Mechanical compartmentalization of the intestinal organoid enables crypt folding and collective cell migration. Nat Cell Biol. 2021;23(7):745–57. 10.1038/s41556-021-00699-6. 34155382 10.1038/s41556-021-00699-6 PMC7611697

[125] Kong X, Wang X, Qin Y, Han J. Effects of sunset yellow on proliferation and differentiation of intestinal epithelial cells in murine intestinal organoids. J Appl Toxicol. 2021;41(6):953–63. 10.1002/jat.4080. 33063357 10.1002/jat.4080

[126] Saheli M, Sepantafar M, Pournasr B, Farzaneh Z, Vosough M, Piryaei A, et al. Three-dimensional liver-derived extracellular matrix hydrogel promotes liver organoids function. J Cell Biochem. 2018;119(6):4320–33. 10.1002/jcb.26622. 29247536 10.1002/jcb.26622

[127] Mun SJ, Ryu JS, Lee MO, Son YS, Oh SJ, Cho HS, et al. Generation of expandable human pluripotent stem cell-derived hepatocyte-like liver organoids. J Hepatol. 2019;71(5):970–85. 10.1016/j.jhep.2019.06.030. 31299272 10.1016/j.jhep.2019.06.030

[128] Zhang N, Ohlstrom D, Pang S, Bharadwaj NS, Qu A, Grossniklaus H, et al. Tissue Spatial Omics Dissects Organoid Biomimicry. GEN Biotechnology. 2023;2(5):372–83. 10.1089/genbio.2023.0039.

[129] Smirnov A, Melino G, Candi E. Gene expression in organoids: an expanding horizon. Biol Direct. 2023;18(1):11. 10.1186/s13062-023-00360-2. 36964575 10.1186/s13062-023-00360-2 PMC10038780

[130] Jansen J, Reimer KC, Nagai JS, Varghese FS, Overheul GJ, de Beer M, et al. SARS-CoV-2 infects the human kidney and drives fibrosis in kidney organoids. Cell Stem Cell. 2022;29(2):217–31. 10.1016/j.stem.2021.12.010. 35032430 10.1016/j.stem.2021.12.010 PMC8709832

[131] Fan YY, Davidson LA, Callaway ES, Wright GA, Safe S, Chapkin RS. A bioassay to measure energy metabolism in mouse colonic crypts, organoids, and sorted stem cells. Am J Physiol Gastrointest Liver Physiol. 2015;309(1):G1-9. 10.1152/ajpgi.00052.2015. 25977509 10.1152/ajpgi.00052.2015 PMC4491508

[132] Inoue M, Tanaka Y, Matsushita S, Shimozaki Y, Ayame H, Akutsu H. Xenogeneic-Free Human Intestinal Organoids for Assessing Intestinal Nutrient Absorption. Nutrients. 2022;14(3). 10.3390/nu14030438. 10.3390/nu14030438 PMC8838315 35276796

[133] Mun SJ, Lee J, Chung KS, Son MY, Son MJ. Effect of Microbial Short-Chain Fatty Acids on CYP3a4-Mediated Metabolic Activation of Human Pluripotent Stem Cell-Derived Liver Organoids. Cells. 2021;10(1). 10.3390/cells10010126. 10.3390/cells10010126 PMC7827634 33440728

[134] Wang Y, Wang H, Deng P, Chen W, Guo Y, Tao T, et al. In situ differentiation and generation of functional liver organoids from human iPSCs in a 3D perfusable chip system. Lab Chip. 2018;18(23):3606–16. 30357207 10.1039/c8lc00869h

[135] Duan X, Tang X, Nair MS, Zhang T, Qiu Y, Zhang W, et al. An airway organoid-based screen identifies a role for the HIF1alpha-glycolysis axis in SARS-CoV-2 infection. Cell Rep. 2021;37(6):109920. 10.1016/j.celrep.2021.109920. 34731648 10.1016/j.celrep.2021.109920 PMC8516798

[136] Han Y, Duan X, Yang L, Nilsson-Payant BE, Wang P, Duan F, et al. Identification of SARS-CoV-2 inhibitors using lung and colonic organoids. Nature. 2021;589(7841):270–5. 10.1038/s41586-020-2901-9. 33116299 10.1038/s41586-020-2901-9 PMC8034380

[137] Miura Y, Li MY, Revah O, Yoon SJ, Narazaki G, Pasca SP. Engineering brain assembloids to interrogate human neural circuits. Nat Protoc. 2022;17(1):15–35. 10.1038/s41596-021-00632-z. 34992269 10.1038/s41596-021-00632-z

[138] Jacques R, Zhou B, Marhuenda E, Gorecki J, Das A, Iskratsch T, et al. Photoelectrochemical imaging of single cardiomyocytes and monitoring of their action potentials through contact force manipulation of organoids. Biosens Bioelectron. 2023;223:115024. 10.1016/j.bios.2022.115024. 36577176 10.1016/j.bios.2022.115024

[139] Kim H, Kamm RD, Vunjak-Novakovic G, Wu JC. Progress in multicellular human cardiac organoids for clinical applications. Cell Stem Cell. 2022;29(4):503–14. 10.1016/j.stem.2022.03.012. 35395186 10.1016/j.stem.2022.03.012 PMC9352318

[140] Beumer J, Geurts MH, Lamers MM, Puschhof J, Zhang J, van der Vaart J, et al. A CRISPR/Cas9 genetically engineered organoid biobank reveals essential host factors for coronaviruses. Nat Commun. 2021;12(1):5498. 10.1038/s41467-021-25729-7. 34535662 10.1038/s41467-021-25729-7 PMC8448725

[141] Zhou J, Li C, Liu X, Chiu MC, Zhao X, Wang D, et al. Infection of bat and human intestinal organoids by SARS-CoV-2. Nat Med. 2020;26(7):1077–83. 10.1038/s41591-020-0912-6. 32405028 10.1038/s41591-020-0912-6

[142] Saul S, Karim M, Ghita L, Huang P, Chiu W, Durán V, et al. Anticancer pan-ErbB inhibitors reduce inflammation and tissue injury and exert broad-spectrum antiviral effects. J Clin Invest. 2023;133(19). 10.1172/JCI169510. 10.1172/JCI169510 PMC10541190 37581931

[143] Al-Tawfiq JA, Al-Homoud AH, Memish ZA. Remdesivir as a possible therapeutic option for the COVID-19. Travel Med Infect Dis. 2020;34:101615. 10.1016/j.tmaid.2020.101615. 32145386 10.1016/j.tmaid.2020.101615 PMC7129391

[144] Joyce MG, Chen WH, Sankhala RS, Hajduczki A, Thomas PV, Choe M, et al. SARS-CoV-2 ferritin nanoparticle vaccines elicit broad SARS coronavirus immunogenicity. Cell Rep. 2021;37(12):110143. 10.1016/j.celrep.2021.110143. 34919799 10.1016/j.celrep.2021.110143 PMC8651551

[145] Pamies D, Ekert J, Zurich MG, Frey O, Werner S, Piergiovanni M, et al. Recommendations on fit-for-purpose criteria to establish quality management for microphysiological systems and for monitoring their reproducibility. Stem Cell Reports. 2024;19(5):604–17. 10.1016/j.stemcr.2024.03.009. 38670111 10.1016/j.stemcr.2024.03.009 PMC11103889

[146] Yang R, Qi Y, Zhang X, Gao H, Yu Y. Living biobank: Standardization of organoid construction and challenges. Chin Med J (Engl). 2024;137(24):3050–60. 10.1097/CM9.0000000000003414. 39663560 10.1097/CM9.0000000000003414 PMC11706585

[147] Phipson B, Er PX, Combes AN, Forbes TA, Howden SE, Zappia L, et al. Evaluation of variability in human kidney organoids. Nat Methods. 2019;16(1):79–87. 10.1038/s41592-018-0253-2. 30573816 10.1038/s41592-018-0253-2 PMC6634992

[148] Wang Y, Li P, Lavrijsen M, Rottier RJ, den Hoed CM, Bruno MJ, et al. Immunosuppressants exert differential effects on pan-coronavirus infection and distinct combinatory antiviral activity with molnupiravir and nirmatrelvir. United European Gastroenterol J. 2023;11(5):431–47. 10.1002/ueg2.12417. 37226653 10.1002/ueg2.12417 PMC10256998

[149] Ianevski A, Froysa IT, Lysvand H, Calitz C, Smura T, Schjelderup NH, et al. The combination of pleconaril, rupintrivir, and remdesivir efficiently inhibits enterovirus infections in vitro, delaying the development of drug-resistant virus variants. Antiviral Res. 2024;224:105842. 10.1016/j.antiviral.2024.105842. 38417531 10.1016/j.antiviral.2024.105842

[150] Kastenschmidt JM, Sureshchandra S, Jain A, Hernandez-Davies JE, de Assis R, Wagoner ZW, et al. Influenza vaccine format mediates distinct cellular and antibody responses in human immune organoids. Immunity. 2023;56(8):1910–26. 10.1016/j.immuni.2023.06.019. 37478854 10.1016/j.immuni.2023.06.019 PMC10433940

[151] Wu CT, Lidsky PV, Xiao Y, Cheng R, Lee IT, Nakayama T, et al. SARS-CoV-2 replication in airway epithelia requires motile cilia and microvillar reprogramming. Cell. 2023;186(1):112–30. 10.1016/j.cell.2022.11.030. 36580912 10.1016/j.cell.2022.11.030 PMC9715480

[152] Jackson CB, Farzan M, Chen B, Choe H. Mechanisms of SARS-CoV-2 entry into cells. Nat Rev Mol Cell Biol. 2022;23(1):3–20. 10.1038/s41580-021-00418-x. 34611326 10.1038/s41580-021-00418-x PMC8491763

[153] Mcmichael AJ, Parham P, Brodsky FM, Pilch JR. Influenza virus-specific cytotoxic T lymphocytes recognize HLA-molecules. Blocking by monoclonal anti-HLA antibodies. J Exp Med. 1980;152(2 Pt 2):195s–203s. 6967937

[154] Ghafari M, Hall M, Golubchik T, Ayoubkhani D, House T, Macintyre-Cockett G, et al. Prevalence of persistent SARS-CoV-2 in a large community surveillance study. Nature. 2024;626(8001):1094–101. 10.1038/s41586-024-07029-4. 38383783 10.1038/s41586-024-07029-4 PMC10901734

[155] Wark P, Pathinayake PS, Eapen MS, Sohal SS. Asthma, COPD and SARS-CoV-2 infection (COVID-19): potential mechanistic insights. Eur Respir J. 2021;58(2). 10.1183/13993003.00920-2021. 10.1183/13993003.00920-2021 PMC8287734 34244320

[156] Puhach O, Meyer B, Eckerle I. SARS-CoV-2 viral load and shedding kinetics. Nat Rev Microbiol. 2023;21(3):147–61. 10.1038/s41579-022-00822-w. 36460930 10.1038/s41579-022-00822-w PMC9716513

[157] Papapetrou EP. Patient-derived induced pluripotent stem cells in cancer research and precision oncology. Nat Med. 2016;22(12):1392–401. 10.1038/nm.4238. 27923030 10.1038/nm.4238 PMC5233709

[158] Bai L, Wu Y, Li G, Zhang W, Zhang H, Su J. AI-enabled organoids: Construction, analysis, and application. Bioact Mater. 2024;31:525–48. 10.1016/j.bioactmat.2023.09.005. 37746662 10.1016/j.bioactmat.2023.09.005 PMC10511344

[159] Li Y, Du J, Deng S, Liu B, Jing X, Yan Y, et al. The molecular mechanisms of cardiac development and related diseases. Signal Transduct Target Ther. 2024;9(1):368. 39715759 10.1038/s41392-024-02069-8 PMC11666744

[160] Foo MA, You M, Chan SL, Sethi G, Bonney GK, Yong WP, et al. Clinical translation of patient-derived tumour organoids- bottlenecks and strategies. Biomark Res. 2022;10(1):10. 35272694 10.1186/s40364-022-00356-6 PMC8908618

[161] Partiot E, Gorda B, Lutz W, Lebrun S, Khalfi P, Mora S, et al. Organotypic culture of human brain explants as a preclinical model for AI-driven antiviral studies. EMBO Mol Med. 2024;16(4):1004–26. 38472366 10.1038/s44321-024-00039-9 PMC11018746

[162] Kumari M, Subbarao N. Deep learning model for virtual screening of novel 3C-like protease enzyme inhibitors against SARS coronavirus diseases. Comput Biol Med. 2021;132:104317. 10.1016/j.compbiomed.2021.104317. 33721736 10.1016/j.compbiomed.2021.104317 PMC7935676

[163] Wang S, Sun Q, Xu Y, Pei J, Lai L. A transferable deep learning approach to fast screen potential antiviral drugs against SARS-CoV-2. Brief Bioinform. 2021;22(6). 10.1093/bib/bbab211. 10.1093/bib/bbab211 PMC8195169 34081143

[164] Zhou G, Rusnac DV, Park H, Canzani D, Nguyen HM, Stewart L, et al. An artificial intelligence accelerated virtual screening platform for drug discovery. Nat Commun. 2024;15(1):7761. 10.1038/s41467-024-52061-7. 39237523 10.1038/s41467-024-52061-7 PMC11377542

[165] Brody H. Influenza Nature. 2019;573(7774):S49. 10.1038/d41586-019-02750-x. 31534258 10.1038/d41586-019-02750-x

[166] Flerlage T, Boyd DF, Meliopoulos V, Thomas PG, Schultz-Cherry S. Influenza virus and SARS-CoV-2: pathogenesis and host responses in the respiratory tract. Nat Rev Microbiol. 2021;19(7):425–41. 10.1038/s41579-021-00542-7. 33824495 10.1038/s41579-021-00542-7 PMC8023351

[167] Taubenberger JK, Morens DM. The pathology of influenza virus infections. Annu Rev Pathol. 2008;3:499–522. 10.1146/annurev.pathmechdis.3.121806.154316. 18039138 10.1146/annurev.pathmechdis.3.121806.154316 PMC2504709

[168] Openshaw P, Chiu C, Culley FJ, Johansson C. Protective and Harmful Immunity to RSV Infection. Annu Rev Immunol. 2017;35:501–32. 10.1146/annurev-immunol-051116-052206. 28226227 10.1146/annurev-immunol-051116-052206

[169] Youk J, Kim T, Evans KV, Jeong YI, Hur Y, Hong SP, et al. Three-Dimensional Human Alveolar Stem Cell Culture Models Reveal Infection Response to SARS-CoV-2. Cell Stem Cell. 2020;27(6):905–19. 10.1016/j.stem.2020.10.004. 33142113 10.1016/j.stem.2020.10.004 PMC7577700

[170] Chiu MC, Li C, Liu X, Song W, Wan Z, Yu Y, et al. Human Nasal Organoids Model SARS-CoV-2 Upper Respiratory Infection and Recapitulate the Differential Infectivity of Emerging Variants. mBio. 2022;13(4):e0194422. 10.1128/mbio.01944-22. 10.1128/mbio.01944-22 PMC9426414 35938726

[171] Porotto M, Ferren M, Chen YW, Siu Y, Makhsous N, Rima B, et al. Authentic Modeling of Human Respiratory Virus Infection in Human Pluripotent Stem Cell-Derived Lung Organoids. mBio. 2019;10(3). 10.1128/mBio.00723-19. 10.1128/mBio.00723-19 PMC6509192 31064833

[172] Chan L, Anderson DE, Cheng HS, Ivan FX, Chen S, Kang A, et al. The establishment of COPD organoids to study host-pathogen interaction reveals enhanced viral fitness of SARS-CoV-2 in bronchi. Nat Commun. 2022;13(1):7635. 10.1038/s41467-022-35253-x. 36496442 10.1038/s41467-022-35253-x PMC9735280

[173] Tsuji S, Minami S, Hashimoto R, Konishi Y, Suzuki T, Kondo T, et al. SARS-CoV-2 infection triggers paracrine senescence and leads to a sustained senescence-associated inflammatory response. Nat Aging. 2022;2(2):115–24. 10.1038/s43587-022-00170-7. 37117754 10.1038/s43587-022-00170-7 PMC10154207

[174] Stroulios G, Brown T, Moreni G, Kondro D, Dei A, Eaves A, et al. Apical-out airway organoids as a platform for studying viral infections and screening for antiviral drugs. Sci Rep. 2022;12(1):7673. 10.1038/s41598-022-11700-z. 35538146 10.1038/s41598-022-11700-z PMC9089294

[175] Chiu MC, Zhang S, Li C, Liu X, Yu Y, Huang J, et al. Apical-Out Human Airway Organoids Modeling SARS-CoV-2 Infection. Viruses. 2023;15(5). 10.3390/v15051166. 10.3390/v15051166 PMC10220522 37243252

[176] Alysandratos KD, Herriges MJ, Kotton DN. Epithelial Stem and Progenitor Cells in Lung Repair and Regeneration. Annu Rev Physiol. 2021;83:529–50. 10.1146/annurev-physiol-041520-092904. 33074772 10.1146/annurev-physiol-041520-092904 PMC9068227

[177] Chiu MC, Li C, Liu X, Yu Y, Huang J, Wan Z, et al. A bipotential organoid model of respiratory epithelium recapitulates high infectivity of SARS-CoV-2 Omicron variant. Cell Discov. 2022;8(1):57. 10.1038/s41421-022-00422-1. 35710786 10.1038/s41421-022-00422-1 PMC9203776

[178] Hook JL, Bhattacharya J. The pathogenesis of influenza in intact alveoli: virion endocytosis and its effects on the lung’s air-blood barrier. Front Immunol. 2024;15:1328453. 10.3389/fimmu.2024.1328453. 38343548 10.3389/fimmu.2024.1328453 PMC10853445

[179] Smith DK, Seales S, Budzik C. Respiratory syncytial virus bronchiolitis in children. Am Fam Physician. 2017;95(2):94–9. 28084708

[180] Matkovic LI, Schneider RT, Thimraj TA, Schrode N, Beitler D, Liu HY, et al. A distal lung organoid model to study interstitial lung disease, viral infection and human lung development. Nat Protoc. 2023;18(7):2283–312. 10.1038/s41596-023-00827-6. 37165073 10.1038/s41596-023-00827-6 PMC11486529

[181] Pezet MG, Torres JA, Thimraj TA, Matkovic I, Schrode N, Murray JW, et al. Human respiratory airway progenitors derived from pluripotent cells generate alveolar epithelial cells and model pulmonary fibrosis. Nat Biotechnol. 2025. 10.1038/s41587-025-02569-0. 10.1038/s41587-025-02569-0 PMC13191444 39994483

[182] Huang J, Hume AJ, Abo KM, Werder RB, Villacorta-Martin C, Alysandratos KD, et al. SARS-CoV-2 Infection of Pluripotent Stem Cell-Derived Human Lung Alveolar Type 2 Cells Elicits a Rapid Epithelial-Intrinsic Inflammatory Response. Cell Stem Cell. 2020;27(6):962–73. 10.1016/j.stem.2020.09.013. 32979316 10.1016/j.stem.2020.09.013 PMC7500949

[183] Tindle C, Fuller M, Fonseca A, Taheri S, Ibeawuchi SR, Beutler N, et al. Adult stem cell-derived complete lung organoid models emulate lung disease in COVID-19. Elife. 2021;10. 10.7554/eLife.66417. 10.7554/eLife.66417 PMC8463074 34463615

[184] Mao L, Jin H, Wang M, Hu Y, Chen S, He Q, et al. Neurologic Manifestations of Hospitalized Patients with Coronavirus Disease 2019 in Wuhan. China JAMA Neurol. 2020;77(6):683–90. 10.1001/jamaneurol.2020.1127. 32275288 10.1001/jamaneurol.2020.1127 PMC7149362

[185] Song E, Zhang C, Israelow B, Lu-Culligan A, Prado AV, Skriabine S, et al. Neuroinvasion of SARS-CoV-2 in human and mouse brain. J Exp Med. 2021;218(3). 10.1084/jem.20202135. 10.1084/jem.20202135 PMC7808299 33433624

[186] Jacob F, Pather SR, Huang WK, Zhang F, Wong S, Zhou H, et al. Human Pluripotent Stem Cell-Derived Neural Cells and Brain Organoids Reveal SARS-CoV-2 Neurotropism Predominates in Choroid Plexus Epithelium. Cell Stem Cell. 2020;27(6):937–50. 10.1016/j.stem.2020.09.016. 33010822 10.1016/j.stem.2020.09.016 PMC7505550

[187] Pellegrini L, Albecka A, Mallery DL, Kellner MJ, Paul D, Carter AP, et al. SARS-CoV-2 Infects the Brain Choroid Plexus and Disrupts the Blood-CSF Barrier in Human Brain Organoids. Cell Stem Cell. 2020;27(6):951–61. 10.1016/j.stem.2020.10.001. 33113348 10.1016/j.stem.2020.10.001 PMC7553118

[188] Wang L, Sievert D, Clark AE, Lee S, Federman H, Gastfriend BD, et al. A human three-dimensional neural-perivascular “assembloid” promotes astrocytic development and enables modeling of SARS-CoV-2 neuropathology. Nat Med. 2021;27(9):1600–6. 10.1038/s41591-021-01443-1. 34244682 10.1038/s41591-021-01443-1 PMC8601037

[189] Wang C, Zhang M, Garcia GJ, Tian E, Cui Q, Chen X, et al. ApoE-Isoform-Dependent SARS-CoV-2 Neurotropism and Cellular Response. Cell Stem Cell. 2021;28(2):331–42. 10.1016/j.stem.2020.12.018. 10.1016/j.stem.2020.12.018 PMC7832490 33450186

[190] Poirier EZ, Buck MD, Chakravarty P, Carvalho J, Frederico B, Cardoso A, et al. An isoform of Dicer protects mammalian stem cells against multiple RNA viruses. Science. 2021;373(6551):231–6. 10.1126/science.abg2264. 34244417 10.1126/science.abg2264 PMC7611482

[191] Miller J, Yan KS. COVID-19 Gastrointestinal Symptoms and Attenuation of the Immune Response to SARS-CoV-2. Gastroenterology. 2021;160(7):2251–4. 10.1053/j.gastro.2021.03.029. 33753106 10.1053/j.gastro.2021.03.029 PMC7973071

[192] Lamers MM, Beumer J, van der Vaart J, Knoops K, Puschhof J, Breugem TI, et al. SARS-CoV-2 productively infects human gut enterocytes. Science. 2020;369(6499):50–4. 10.1126/science.abc1669. 32358202 10.1126/science.abc1669 PMC7199907

[193] Kruger J, Gross R, Conzelmann C, Muller JA, Koepke L, Sparrer K, et al. Drug Inhibition of SARS-CoV-2 Replication in Human Pluripotent Stem Cell-Derived Intestinal Organoids. Cell Mol Gastroenterol Hepatol. 2021;11(4):935–48. 10.1016/j.jcmgh.2020.11.003. 33186749 10.1016/j.jcmgh.2020.11.003 PMC7655023

[194] Prelli BC, Nchioua R, Volcic M, Koepke L, Kruger J, Schutz D, et al. IFITM proteins promote SARS-CoV-2 infection and are targets for virus inhibition in vitro. Nat Commun. 2021;12(1):4584. 10.1038/s41467-021-24817-y. 34321474 10.1038/s41467-021-24817-y PMC8319209

[195] Stanifer ML, Kee C, Cortese M, Zumaran CM, Triana S, Mukenhirn M, et al. Critical Role of Type III Interferon in Controlling SARS-CoV-2 Infection in Human Intestinal Epithelial Cells. Cell Rep. 2020;32(1):107863. 10.1016/j.celrep.2020.107863. 32610043 10.1016/j.celrep.2020.107863 PMC7303637

[196] Triana S, Metz-Zumaran C, Ramirez C, Kee C, Doldan P, Shahraz M, et al. Single-cell analyses reveal SARS-CoV-2 interference with intrinsic immune response in the human gut. Mol Syst Biol. 2021;17(4):e10232. 10.15252/msb.202110232. 10.15252/msb.202110232 PMC8077299 33904651

[197] Jothimani D, Venugopal R, Abedin MF, Kaliamoorthy I, Rela M. COVID-19 and the liver. J Hepatol. 2020;73(5):1231–40. 10.1016/j.jhep.2020.06.006. 32553666 10.1016/j.jhep.2020.06.006 PMC7295524

[198] Mccarron S, Bathon B, Conlon DM, Abbey D, Rader DJ, Gawronski K, et al. Functional Characterization of Organoids Derived From Irreversibly Damaged Liver of Patients with NASH. Hepatology. 2021;74(4):1825–44. 10.1002/hep.31857. 33901295 10.1002/hep.31857 PMC12928191

[199] Xia S, Wu M, Chen S, Zhang T, Ye L, Liu J, et al. Long Term Culture of Human Kidney Proximal Tubule Epithelial Cells Maintains Lineage Functions and Serves as an Ex vivo Model for Coronavirus Associated Kidney Injury. Virol Sin. 2020;35(3):311–20. 10.1007/s12250-020-00253-y. 32602046 10.1007/s12250-020-00253-y PMC7322379

[200] Monteil V, Kwon H, Prado P, Hagelkruys A, Wimmer RA, Stahl M, et al. Inhibition of SARS-CoV-2 Infections in Engineered Human Tissues Using Clinical-Grade Soluble Human ACE2. Cell. 2020;181(4):905–13. 10.1016/j.cell.2020.04.004. 32333836 10.1016/j.cell.2020.04.004 PMC7181998

[201] Delorey TM, Ziegler C, Heimberg G, Normand R, Yang Y, Segerstolpe A, et al. COVID-19 tissue atlases reveal SARS-CoV-2 pathology and cellular targets. Nature. 2021;595(7865):107–13. 10.1038/s41586-021-03570-8. 33915569 10.1038/s41586-021-03570-8 PMC8919505

[202] Brauninger H, Stoffers B, Fitzek A, Meissner K, Aleshcheva G, Schweizer M, et al. Cardiac SARS-CoV-2 infection is associated with pro-inflammatory transcriptomic alterations within the heart. Cardiovasc Res. 2022;118(2):542–55. 10.1093/cvr/cvab322. 34647998 10.1093/cvr/cvab322 PMC8803085

[203] Thomas D, Noishiki C, Gaddam S, Wu D, Manhas A, Liu Y, et al. CCL2-mediated endothelial injury drives cardiac dysfunction in long COVID. Nat Cardiovasc Res. 2024;3(10):1249–65. 10.1038/s44161-024-00543-8. 39402206 10.1038/s44161-024-00543-8 PMC12243935

[204] Esch EW, Bahinski A, Huh D. Organs-on-chips at the frontiers of drug discovery. Nat Rev Drug Discov. 2015;14(4):248–60. 10.1038/nrd4539. 25792263 10.1038/nrd4539 PMC4826389

[205] Thacker VV, Sharma K, Dhar N, Mancini GF, Sordet-Dessimoz J, Mckinney JD. Rapid endotheliitis and vascular damage characterize SARS-CoV-2 infection in a human lung-on-chip model. EMBO Rep. 2021;22(6):e52744. 10.15252/embr.202152744. 10.15252/embr.202152744 PMC8183417 33908688

[206] Si L, Bai H, Rodas M, Cao W, Oh CY, Jiang A, et al. A human-airway-on-a-chip for the rapid identification of candidate antiviral therapeutics and prophylactics. Nat Biomed Eng. 2021;5(8):815–29. 10.1038/s41551-021-00718-9. 33941899 10.1038/s41551-021-00718-9 PMC8387338

[207] Utzon AN, Johansen IS, Bang LL, Pedersen RM, Andersen TE, Madsen LW. Viral dynamics of SARS-CoV-2 in immunocompromised patients. Clin Microbiol Infect. 2023;29(8):1081–7. 10.1016/j.cmi.2023.05.013. 10.1016/j.cmi.2023.05.013 PMC10181867 37182645

[208] Woodall M, Cujba AM, Worlock KB, Case KM, Masonou T, Yoshida M, et al. Age-specific nasal epithelial responses to SARS-CoV-2 infection. Nat Microbiol. 2024;9(5):1293–311. 10.1038/s41564-024-01658-1. 38622380 10.1038/s41564-024-01658-1 PMC11087271

[209] Lamers MM, Haagmans BL. SARS-CoV-2 pathogenesis. Nat Rev Microbiol. 2022;20(5):270–84. 10.1038/s41579-022-00713-0. 35354968 10.1038/s41579-022-00713-0

[210] Koike H, Iwasawa K, Ouchi R, Maezawa M, Giesbrecht K, Saiki N, et al. Modelling human hepato-biliary-pancreatic organogenesis from the foregut-midgut boundary. Nature. 2019;574(7776):112–6. 10.1038/s41586-019-1598-0. 31554966 10.1038/s41586-019-1598-0 PMC7643931

[211] Ronaldson-Bouchard K, Teles D, Yeager K, Tavakol DN, Zhao Y, Chramiec A, et al. A multi-organ chip with matured tissue niches linked by vascular flow. Nat Biomed Eng. 2022;6(4):351–71. 35478225 10.1038/s41551-022-00882-6 PMC9250010

[212] Marx U, Akabane T, Andersson TB, Baker E, Beilmann M, Beken S, et al. Biology-inspired microphysiological systems to advance patient benefit and animal welfare in drug development. ALTEX. 2020;37(3):365–94. 10.14573/altex.2001241. 10.14573/altex.2001241 PMC7863570 32113184

